# Supplementary material for: Differential Sperm Motility Mediates the Sex Ratio Drive Shaping Mouse Sex Chromosome Evolution
Source: Curr Biol. 2019 Nov 4;29(21):3692–3698.e4. doi: 10.1016/j.cub.2019.09.031 (PMC6839398; doi:10.1016/j.cub.2019.09.031)
Supplement: Document S1. Figures S1–S4 and Tables S1–S4 [file mmc1.pdf]

Current Biology, Volume *29*

Supplemental Information

Differential Sperm Motility Mediates the Sex Ratio Drive Shaping Mouse Sex Chromosome Evolution

Claudia Cattoni Rathje, Emma Elizabeth Philippa Johnson, Deborah Drage, Christina Patinioti, Giuseppe Silvestri, Nabeel Ahmed Affara, Côme Ialy-Radio, Julie Cocquet, Benjamin Matthew Skinner, and Peter James Ivor Ellis

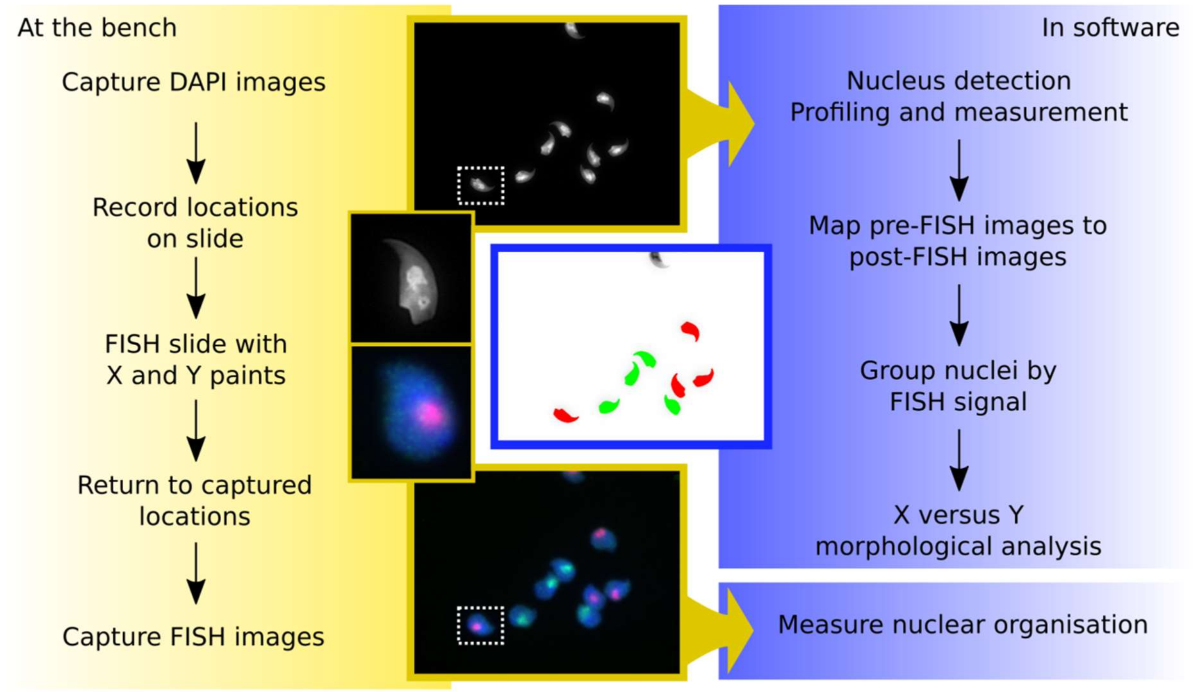

Figure S1. Flowchart showing the capture-recapture protocol. Related STAR Methods. Flowchart showing the capture-recapture protocol. The extensive swelling required for FISH probes to penetrate the nucleus (inset nuclei show before and after FISH) requires storing slide coordinates and imaging the slide before and after FISH for morphology analysis and subsequent XY grouping. A software user interface allows simple assignment of pre-FISH nuclei based on post-FISH images.

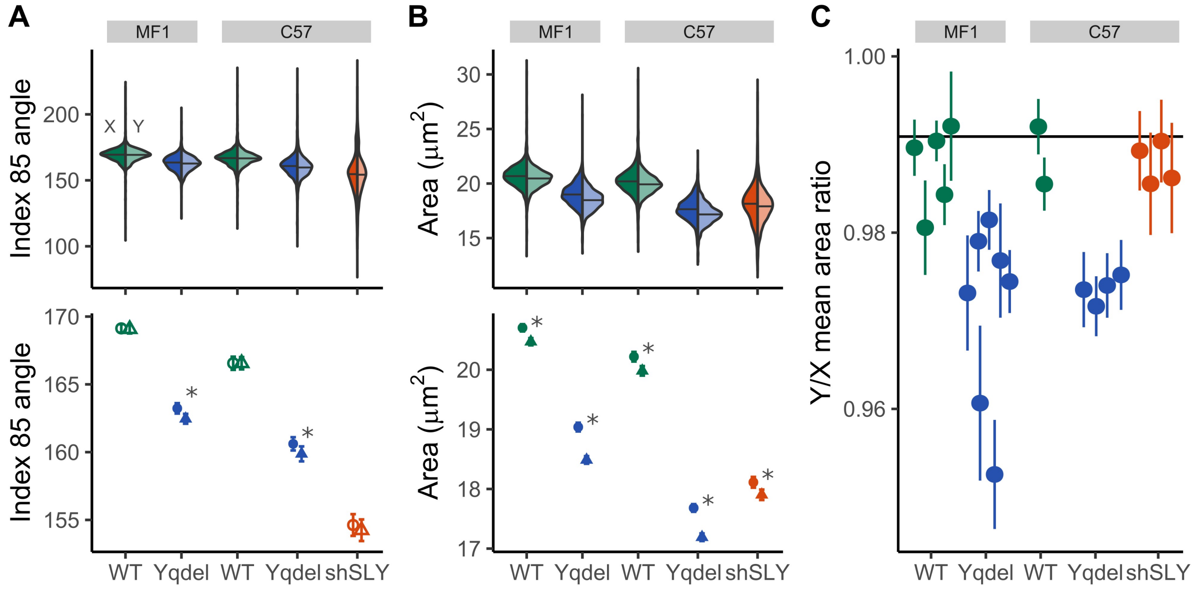

Figure S2. Detailed differences in sperm shape and size. Related to Figure 2. A) Angles at profile index 85 in each strain, comparing X- and Y-bearing sperm. The upper panel shows violin plots of the complete population data for each strain, while the lower panel shows the mean and standard error of the mean in order to highlight differences. Significant XY differences (Mann-Whitney test, $p < 0.05$) are marked '*'. Y-bearing sperm have a significantly more curved acrosome in Yqdel animals only. B) Comparison of nuclear areas, using the same format as (A); Y-bearing sperm are consistently smaller than X-bearing sperm in all genotypes including wild types. C) Comparison of the mean Y/X area ratio in each sample (see Table S1 for the details of sample numbers analysed per genotype), with standard error of the mean. The horizontal line indicates the mean wild type ratio. Wild type and shSLY samples have similar area ratios, while Yqdel samples show a further reduction in Y area.

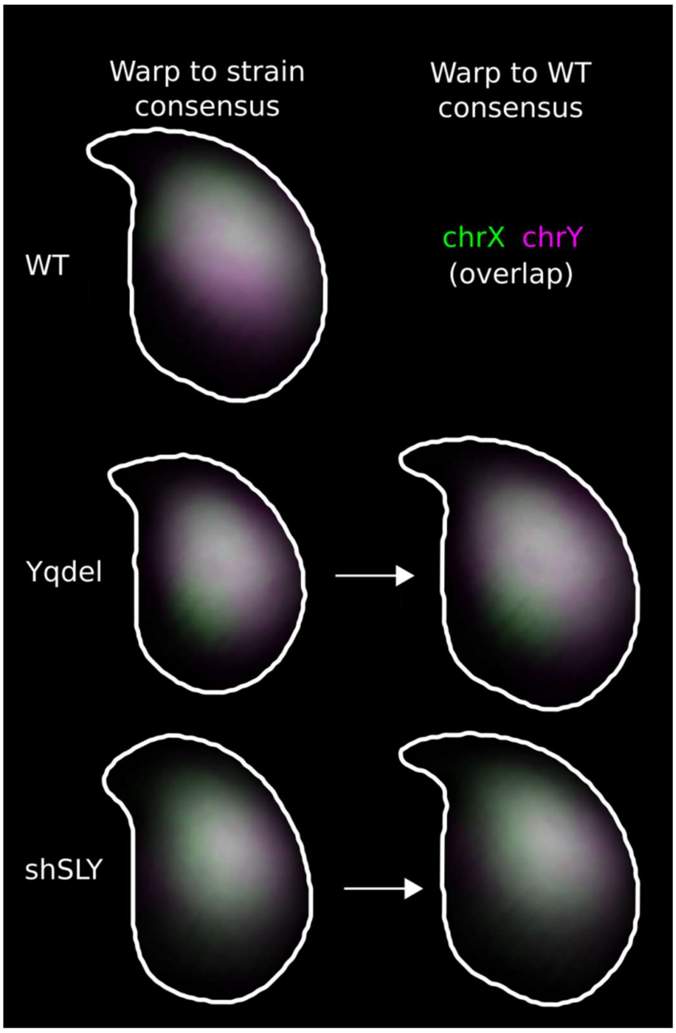

Figure S3. Comparison of chromosome positions in WT versus Yq-del sperm. Related to Figure 2. Chromosome territories for X and Y are sub-acrosomal in C57Bl6, and strongly overlap in all genotypes analysed (MS-SSIM* 0.86-0.94). Following warping to a WT template, the location of the X and Y chromosomes in sperm from Yqdel and shSLY males closely resembles their locations in wild type C57Bl6 sperm (MS-SSIM* 0.82-0.86). This indicates morphological changes are external to the nucleus, and do not involve other chromatin reorganisation.

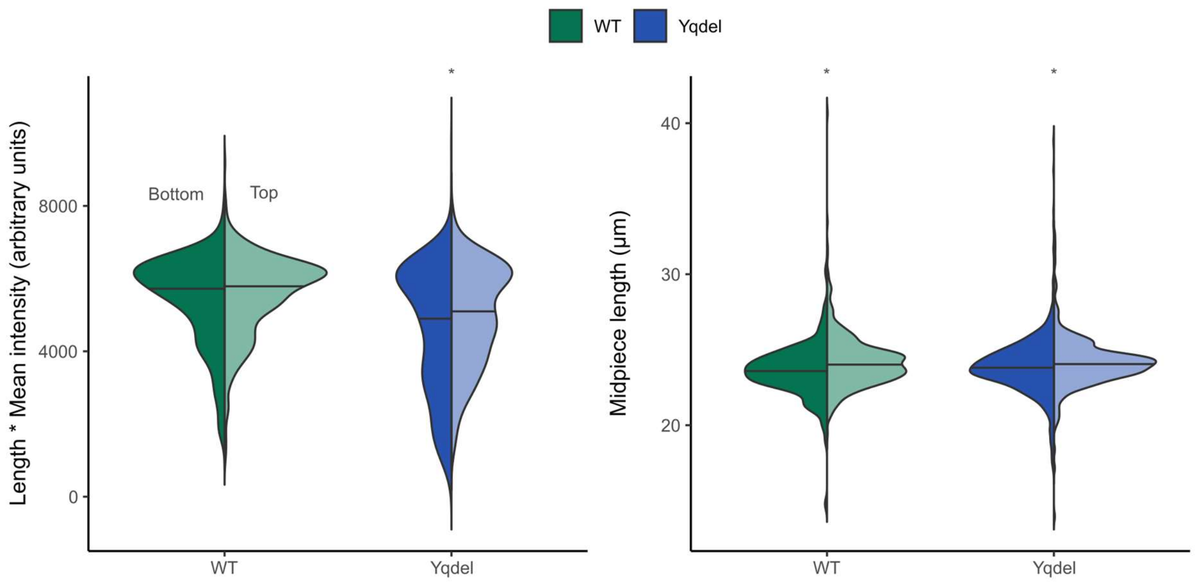

Figure S4. Poorer swimming sperm have lower mitochondrial activity. Related to Figure 4. Midpiece length multiplied by mean signal is a proxy for total mitochondrial activity. Horizontal lines show sample medians. A two-sample two-tailed Kolmogorov-Smirnov test detects no difference between top and bottom for multiplied values in wild type XY^{RIII} (p=0.73) and a significant difference in XY^{RIII}qdel (p=7.15E-05). Midpiece length is a proxy for mitochondrial count, and is slightly higher in top fractions in both XY^{RIII} (p=0.0004) and XY^{RIII}qdel (p=0.006).

Protocol	Paternal genotype	Number of males	Sperm status	Maternal details	Number of offspring	Percent female offspring	S.E.P.
Colony data (Cambs)	XYRIIIqdel	--	Pair or trio mating cages	Predominantly MF1	723	54.9%	1.85%
Colony data (Cambs)	XYRIII	--	Pair or trio mating cages	Predominantly MF1	922	47.2%	1.64%
Colony data (Kent)	XYRIIIqdel	--	Pair or trio mating cages	MF1	526	58.9%	2.15%
Colony data (Kent)	XYRIII	--	Pair or trio mating cages	MF1	534	45.7%	2.16%
Colony data (France)	XYRIIIqdel	--	Pair or trio mating cages	>80% C57Bl6/N	132	61.4%	4.24%
Colony data (France)	XYRIII	--	Pair or trio mating cages	>80% C57Bl6/N	151	51.7%	4.07%
Colony data (France)	shSLY	--	Pair or trio mating cages	>80% C57Bl6/N	16	87.5%	8.27%
IVF, GFP scoring of blastocysts	XGFPYRIIIqdel	6	cauda epididymal sperm retrieved for IVF	CBAB6 F1 oocytes (cumulus retained)	243	44.4%	3.19%
				CBAB6 F1 oocytes (cumulus removed)	203	44.8%	3.49%
				Total:	446	44.6%	2.35%
Superovulation, natural mating, 2-cell embryo recovery, GFP scoring of blastocysts	XGFPYRIIIqdel	9	fresh (3 day mating interval)	CBAB6 F1	173	55.5%	3.78%
			aged (14+ day mating interval)	CBAB6 F1	251	53.8%	3.15%

		4	aged (14+ day mating interval)	MF1	87	60.9%	5.23%
				Total:	511	55.6%	2.20%
Superovulation, natural mating, 2-cell embryo recovery, GFP scoring of blastocysts	XGFPYRIII	9	fresh (3 day mating interval)	CBAB6 F1	150	50.0%	4.08%
			aged (14+ day mating interval)	CBAB6 F1	104	47.1%	4.89%
		4	aged (14+ day mating interval)	MF1	73	38.4%	5.69%
				Total:	327	46.5%	2.76%
Natural mating (timed oestrus), GFP scoring of mid-gestation embryos	XGFPYRIIIqdel	7	~7 day mating interval	XYRIII daughters	143	58.0%	4.13%
			~7 day mating interval	XYRIIIqdel daughters	159	54.7%	3.95%
				Total:	302	56.3%	2.85%
Natural mating (timed oestrus), GFP scoring of mid-gestation embryos	XGFPYRIII	7	~7 day mating interval	XYRIII daughters	111	43.2%	4.70%
			~7 day mating interval	XYRIIIqdel daughters	143	48.3%	4.18%
				Total:	254	46.1%	3.13%

Table S1. Offspring sex ratios in animals in this study. Related to Figure 1. Offspring sex ratios shift in favour of females under natural mating in animals carrying the Y^{RIII}qdel chromosome compared to animals carrying the Y^{RIII} chromosome when using both fresh and aged sperm; this difference is abolished by IVF using embryos with or without cumulus cells (see also **Figure 1**). S.E.P, standard error of proportion.

		WT		Yqdel		shSLY	
**Background**	**Cluster**	**#X / #Y sperm**	**%X ± S.E.P.**	**#X / #Y sperm**	**%X ± S.E.P.**	**#X / #Y sperm**	**%X ± S.E.P.**
MF1	N1	995 / 946	51.3 ± 1.1%	68 / 47	59.1 ± 4.6%	-	-
	N2	1114 / 1022	52.2 ± 1.1%	135 / 113	54.4 ± 3.2%	-	-
	A1	35 / 24	59.3 ± 6.4%	1015 / 1053	49.1 ± 1.1%	-	-
	A2	53 / 49	52 ± 4.9%	420 / 568	42.5 ± 1.6%	-	-
	**Total**	**2197 / 2041**	**51.8 ± 0.8%**	**1638 / 1781**	**47.9 ± 0.9%**	-	-
C57Bl6	N	969 / 961	50.2 ± 1.1%	65 / 47	58 ± 4.7%	2 / 1	66.7 ± 27.2%
	A1	58 / 46	55.8 ± 4.9%	808 / 776	51 ± 1.3%	33 / 26	55.9 ± 6.5%
	A2	20 / 21	48.8 ± 7.8%	190 / 217	46.7 ± 2.5%	198 / 161	55.2 ± 2.6%
	A3	17 / 18	48.6 ± 8.4%	47 / 48	49.5 ± 5.1%	943 / 963	49.5 ± 1.1%
	B1	194 / 189	50.7 ± 2.6%	374 / 356	51.2 ± 1.9%	346 / 321	51.9 ± 1.9%
	B2	104 / 119	46.6 ± 3.3%	220 / 203	52 ± 2.4%	290 / 295	49.6 ± 2.1%
	S	30 / 30	50 ± 6.5%	155 / 122	56 ± 3%	669 / 690	49.2 ± 1.4%
	**Total**	**1392 / 1384**	**50.1 ± 0.1%**	**1859 / 1769**	**51.2 ± 0.1%**	**2481 / 2457**	**50.2 ± 0.1%**

Table S2. Number and proportion of X- and Y-bearing sperm in each morphological cluster. Related to Figure 3. On an MF1 background, more abnormal sperm are enriched for Y-bearing sperm (Fisher's exact test, p=0.002) in Yqdel samples, but not in WT samples (p=1). On the C57Bl6 background, a similar gradient is observed in Yqdel and shSLY samples, but is not statistically significant in these (p=0.63, 0.66), or in WT (p=1). S.E.P, standard error of proportion.

Type	Sample	Number of animals	Ages (weeks)	n cells	X	Y	Average X area	Average Y area	Y/X area ratio	Area difference	S.E.M.	Volume difference	S.E.M.
**MF1 WT**	WT P1	8	13 - 16	1219	634	585	20.78	20.56	99.0%	1.0%	0.3%	1.6%	0.5%
	WT P2	8	10 - 14	777	426	351	20.37	20.05	98.4%	1.6%	0.3%	2.3%	0.5%
	WT I1	1	10.9	1524	777	747	20.93	20.73	99.0%	1.0%	0.2%	1.4%	0.3%
	WT I2	1	29.7	305	150	155	20.76	20.59	99.2%	0.8%	0.6%	1.2%	0.9%
	WT I3	1	26.1	413	210	203	20.28	19.88	98.1%	1.9%	0.5%	2.9%	0.8%
	Average						*20.62*	*20.36*	*98.7%*	*1.3%*	*0.2%*	*1.9%*	*0.3%*
	**Aggregate**			4238	2197	2041	**20.70**	**20.47**	**98.9%**	**1.1%**	**0.2%**	**1.7%**	**0.2%**
**MF1 Yqdel**	Yqdel P1	5	11 - 14.5	904	386	518	18.50	18.02	97.4%	2.6%	0.4%	3.8%	0.5%
	Yqdel P2	6	9.3	308	119	189	18.93	18.03	95.3%	4.7%	0.6%	7.0%	0.9%
	Yqdel I1	1	11	860	440	420	19.76	19.35	97.9%	2.1%	0.3%	3.1%	0.5%
	Yqdel I2	1	11	262	136	126	18.79	18.35	97.7%	2.3%	0.6%	3.5%	1.0%
	Yqdel I3	1	11	574	279	295	18.93	18.58	98.1%	1.9%	0.3%	2.8%	0.5%
	Yqdel I4	1	31.3	177	91	86	19.31	18.55	96.1%	3.9%	0.9%	5.8%	1.3%
	Yqdel I5	1		187	94	93	18.44	17.94	97.3%	2.7%	0.7%	4.0%	1.0%

	Average						*18.95*	*18.40*	*97.1%*	*2.9%*	*0.4%*	*4.3%*	*0.5%*
	**Aggregate**			3419	1638	1781	**19.01**	**18.49**	**97.3%**	**2.7%**	**0.2%**	**4.1%**	**0.3%**
**C57Bl6 WT**	WT 1	1	24.6	1541	784	757	20.30	20.01	98.6%	1.4%	0.3%	2.2%	0.5%
	WT 2	1	24.6	1235	608	627	20.11	19.95	99.2%	0.8%	0.3%	1.2%	0.5%
	Average						*20.21*	*19.98*	*98.9%*	*1.1%*	*0.8%*	*1.7%*	*1.1%*
	**Aggregate**			2776	1392	1384	**20.22**	**19.98**	**98.8%**	**1.2%**	**0.2%**	**1.8%**	**0.3%**
**C57Bl6 Yqdel**	Yqdel 1	1	14.6	1102	566	536	17.94	17.47	97.4%	2.6%	0.4%	3.9%	0.5%
	Yqdel 2	1	14.6	1101	594	507	17.87	17.36	97.2%	2.8%	0.3%	4.2%	0.5%
	Yqdel 3	1	11.4	690	344	346	17.60	17.14	97.4%	2.6%	0.4%	3.9%	0.6%
	Yqdel 4	1	11.4	735	355	380	17.04	16.62	97.5%	2.5%	0.4%	3.7%	0.6%
	Average						*17.61*	*17.15*	*97.4%*	*2.6%*	*0.1%*	*3.9%*	*0.1%*
	**Aggregate**			3628	1859	1769	**17.68**	**17.19**	**97.2%**	**2.8%**	**0.2%**	**4.1%**	**0.3%**
**C57Bl6 shSLY**	shSLY 1	1	14.9	1149	587	562	17.89	17.65	98.6%	1.4%	0.6%	2.1%	0.9%
	shSLY 2	1	24.6	1261	653	608	17.84	17.65	98.9%	1.1%	0.5%	1.6%	0.7%
	shSLY 3	1	24.6	1368	666	702	17.99	17.81	99.0%	1.0%	0.5%	1.4%	0.7%

	shSLY 4	1	24.6	1160	575	585	18.80	18.53	98.6%	1.4%	0.6%	2.2%	0.9%
	Average						*18.13*	*17.91*	*98.8%*	*1.2%*	*0.1%*	*1.8%*	*0.2%*
	**Aggregate**			4938	2481	2457	**18.11**	**17.90**	**98.8%**	**1.2%**	**0.3%**	**1.7%**	**0.4%**

Table S3. Details of the individual samples analysed, and the measured size differences in X- and Y- bearing sperm. Related to Figure 2 and Figure 3. Details of the individual samples analysed, and the measured size differences in X- and Y- bearing sperm. S.E.M, standard error of the mean. The volume difference was calculated from area, assuming an equivalent reduction in sperm thickness.

	Estimate	Std. Error	z value	Pr(>|z|)
(Intercept)	0.015374	0.018551	0.829	0.407
Segment	-0.010992	0.004764	-2.307	0.021
Strain alone	0.038406	0.026236	1.464	0.143
Live/dead state	-0.142011	0.024665	-5.758	8.53E-09
Segment:Strain	-0.001866	0.006737	-0.277	0.782
Segment:State	0.032999	0.006333	5.211	1.88E-07
Strain:State	0.231879	0.034883	6.647	2.98E-11
Segment:Strain:State	-0.049502	0.008956	-5.527	3.25E-08

Table S4. Summary of the beta regression on swim-up data, showing the significance of each parameter and their interactions. Related to Figure 4. Summary of the beta regression on swim-up data, showing the significance of each parameter and their interactions. The pseudo R^2 was 0.87.
