# Supplementary material for: Differential Sperm Motility Mediates the Sex Ratio Drive Shaping Mouse Sex Chromosome Evolution
Source: Curr Biol. 2019 Nov 4;29(21):3692–3698.e4. doi: 10.1016/j.cub.2019.09.031 (PMC6839398; doi:10.1016/j.cub.2019.09.031)
Supplement: Document S2. Article plus Supplemental Information [file mmc2.pdf]

Report

Current Biology

Differential Sperm Motility Mediates the Sex Ratio Drive Shaping Mouse Sex Chromosome Evolution

Highlights

- The sex ratio skew in the offspring of Yq-deleted male mice is abolished by IVF
- In Yqdel males, Y sperm are more severely morphologically distorted than X sperm
- Similarly, Y sperm in these males have relatively impaired motility
- This motility difference explains the sex ratio skew in offspring of these males

Authors

Claudia Cattoni Rathje, Emma Elizabeth Philippa Johnson, Deborah Drage, ..., Julie Cocquet, Benjamin Matthew Skinner, Peter James Ivor Ellis

Correspondence

p.j.i.ellis@kent.ac.uk

In Brief

Mouse sex chromosome evolution has been shaped by widespread gene amplification, due to genomic conflict over offspring sex ratio. Rathje et al. show that male mice with a partial Y chromosome deletion show impaired morphology and motility of Y- versus X-bearing sperm, revealing the physiological basis of the sex ratio drive underpinning the conflict.

Rathje et al., 2019, Current Biology *29*, 3692–3698
November 4, 2019 © 2019 The Authors. Published by Elsevier Ltd.
https://doi.org/10.1016/j.cub.2019.09.031

Current Biology
Report

Differential Sperm Motility Mediates the Sex Ratio Drive Shaping Mouse Sex Chromosome Evolution

Claudia Cattoni Rathje,[1] Emma Elizabeth Philippa Johnson,[2] Deborah Drage,[3] Christina Patinioti,[1] Giuseppe Silvestri,[1] Nabeel Ahmed Affara,[2] Côme Ialy-Radio,[4,5,6] Julie Cocquet,[4,5,6] Benjamin Matthew Skinner,[2,7,8] and Peter James Ivor Ellis[1,8,9,*]
[1]School of Biosciences, University of Kent, Canterbury CT2 7NJ, UK
[2]Department of Pathology, University of Cambridge, Tennis Court Road, Cambridge CB2 1QP, UK
[3]University Biomedical Services, University of Cambridge, Cambridge CB2 2SP, UK
[4]Department of Development, Reproduction and Cancer, INSERM, U1016, Institut Cochin, Paris, France
[5]CNRS, UMR8104, Paris, France
[6]Sorbonne Paris Cité, Faculté de Médecine, Université Paris Descartes, Paris, France
[7]School of Life Sciences, University of Essex, Wivenhoe Park, Colchester CO4 3SQ, UK
[8]These authors contributed equally
[9]Lead Contact
*Correspondence: p.j.i.ellis@kent.ac.uk
https://doi.org/10.1016/j.cub.2019.09.031

SUMMARY

The mouse sex chromosomes exhibit an extraordinary level of copy number amplification of postmeiotically expressed genes [1, 2], driven by an "arms race" (genomic conflict) between the X and Y chromosomes over the control of offspring sex ratio. The sex-linked ampliconic transcriptional regulators *Slx* and *Sly* [3–7] have opposing effects on global transcription levels of the sex chromosomes in haploid spermatids via regulation of postmeiotic sex chromatin (PMSC) [8–11] and opposing effects on offspring sex ratio. Partial deletions of the Y chromosome (Yq) that reduce *Sly* copy number lead to global overexpression of sex-linked genes in spermatids and either a distorted sex ratio in favor of females (smaller deletions) or sterility (larger deletions) [12–16]. Despite a large body of work studying the role of the sex chromosomes in regulating spermatogenesis (recent reviews [17–20]), most studies do not address differential fertility effects on X- and Y-bearing cells. Hence, in this study, we concentrate on identifying physiological differences between X- and Y-bearing sperm from Yq-deleted males that affect their relative fertilizing ability and consequently lead to sex ratio skewing. We show that X- and Y-bearing sperm in these males have differential motility and morphology but are equally able to penetrate the cumulus and fertilize the egg once at the site of fertilization. The altered motility is thus deduced to be the proximate cause of the skew. This represents the first demonstration of a specific difference in sperm function associated with sex ratio skewing.

RESULTS

In Vitro Fertilization (IVF) Abolishes Offspring Sex Ratio Skewing for MF1-XY^{RIII}qdel Males

Intracytoplasmic sperm injection (ICSI) was previously shown to abolish the skew, showing that Yq-deleted males produce equal numbers of X- and Y-bearing sperm that are functionally different from each other [21]. Consequently, we focused first on mechanisms through which X and Y sperm may differ in their ability to enter the oocyte. Based on existing data showing an interaction between sex chromosome complement and transmission ratio distortion mediated by *Spam1* hyaluronidase deficiency in males heterozygous for Robertsonian (Rb) fusions involving chromosome 6 [22–25], our initial hypothesis was that the sex ratio skew in Yq-deleted males was related to differential penetration of the cumulus cell complex surrounding the oocyte and that removal of the cumulus would reduce or abolish the sex ratio skew.

To test this, we focused on an XY^{RIII} partial Yq deletion [13] that in our colonies leads to at least a 7.7 percentage point difference in sex ratio (Cambridge colony on outbred MF1 background, 47.2% females for control XY^{RIII} males and 54.9% females for XY^{RIII}qdel males, $p = 0.002$; Kent colony on outbred MF1 background, 45.7% females for control XY^{RIII} males and 58.9% females for XY^{RIII}qdel males, $p < 0.001$; Paris colony on inbred C57Bl6/N background, 50.1% females for control XY^{RIII} males, 61.4% females for XY^{RIII}qdel males, 87.5% females for XY^{RIII}-shSLY males, $p = 0.01$; all statistics two-tailed two/three-proportions Z-test). In our Cambridge colony, we introduced an X-linked GFP transgene [26] to allow genotyping of preimplantation embryos. Using sperm from the resulting X^{GFP}Y^{RIII}qdel males, we generated matched sets of IVF offspring with and without hyaluronidase pre-treatment of the oocytes to remove cumulus cells ($n = 6$ males tested). Surprisingly, the female skew was abolished in both the cumulus-on and cumulus-off experimental groups, with both instead showing a slight male bias consistent with the strain background sex ratio (Figure 1; Table S1).

 © 2019 The Authors. Published by Elsevier Ltd.
This is an open access article under the CC BY license (http://creativecommons.org/licenses/by/4.0/).

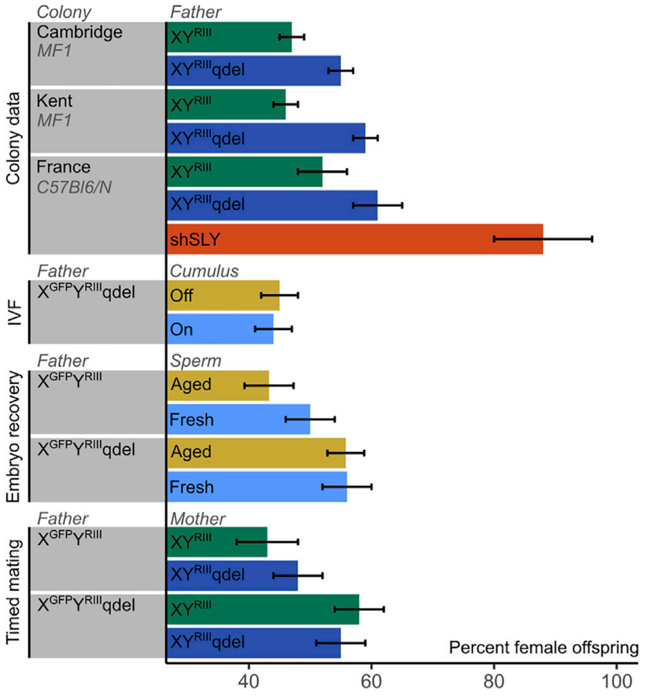

Figure 1. Sex Ratios Observed in Colony Mating and in Embryos Generated by IVF versus Natural Mating and Scored at Differing Time Points

See also Table S1 for raw counts. XY^{RIII}qdel animals and $X^{GFP}Y^{RIII}$qdel embryos show a marked sex ratio skew in favor of females when mated naturally, but this is abolished in IVF experiments. XY^{RIII}qdel animals and $X^{GFP}Y^{RIII}$ and IVF-derived $X^{GFP}Y^{RIII}$qdel embryos show a slight skew in favor of males. Error bars show SE of proportion.

Next, to test whether the abolition of the sex ratio skew in the IVF cohorts was due to the fertilization procedure and not an artifact of the embryo culture step, we mated $X^{GFP}Y^{RIII}$qdel and control $X^{GFP}Y^{RIII}$ males (n = 9 males tested per genotype) to superovulated females to allow natural fertilization, collected the resulting embryos at the 2-cell stage by flushing oviducts at 1.5 days post coitus, and cultured them *in vitro* to blastocyst stage for GFP scoring. In this experiment, we also took the opportunity to test whether the sex ratio skew was affected by epididymal aging in either $X^{GFP}Y^{RIII}$qdel or control males by varying the inter-mating interval for the fathers (STAR Methods). We observed no difference between fresh and aged sperm for either genotype, but there was a significant skew in favor of females in the offspring of $X^{GFP}Y^{RIII}$qdel males (combining aged and fresh sperm data; two-tailed binomial $p = 0.0113$ relative to null expectation of 50:50 ratio; $p = 0.0102$ relative to $X^{GFP}Y^{RIII}$ control data; $p = 0.0007$ relative to $X^{GFP}Y^{RIII}$qdel IVF data; Table S1).

Finally, given the documented imprinted effects of Yq deletion on the cumulus cell properties of daughters of B10.BR-Y^{del} males [27], we tested whether there was an imprinted effect on the sex ratio skew by mating $X^{GFP}Y^{RIII}$qdel and $X^{GFP}Y^{RIII}$ males (n = 7 males tested per genotype) to daughters of XY^{RIII}qdel or XY^{RIII} males and scoring the resulting offspring. In this experiment, the females were not superovulated and the embryos were dissected and scored for GFP in mid-gestation. Although there was again a significant skew toward females in the offspring of $X^{GFP}Y^{RIII}$qdel males (two-tailed binomial $p = 0.0285$ relative to null expectation of 50:50 ratio; $p = 0.0162$ relative to MF1-$X^{GFP}Y^{RIII}$ control males), there was no effect of the maternal background.

Yq Deletion Affects Y-Bearing Sperm Morphology More Severely Than X-Bearing Sperm

The female tract has been shown to discriminate between morphologically normal B10.BR sperm versus abnormal B10.BR-Y^{del} sperm at the uterotubular junction [28]. If Y-bearing sperm are more severely morphologically distorted than X-bearing sperm in Yq-deleted males, this might impair their ability to pass this junction. This in turn would enrich for X-bearing sperm among the population reaching the site of fertilization in the oviduct. We therefore systematically tested for morphological differences between X- and Y-bearing sperm, using a novel image analysis tool for quantitative sperm morphometry [29, 30], and a repeat-imaging protocol (Figure S1) that allowed us to capture pre-fluorescence *in situ* hybridization (FISH) morphology data and correlate this with post-FISH identification of X- versus Y-bearing status.

We analyzed the effect of Yq deletion on both MF1 and C57Bl6 genetic backgrounds, using animals from all three of our colonies. This allowed us to also analyze shSLY males, which are maintained on a C57Bl6 background [3]. shSLY males exhibit a similar degree of offspring sex ratio skewing to Yq-deleted males but a much higher level of severe sperm malformation and impaired fertility. Importantly, shSLY males have no loss of Y chromosomal DNA, allowing us to test whether any morphological differences between X- and Y-bearing sperm are a consequence of the reduction in DNA content in the Yq-deleted sperm. On both genetic backgrounds, our morphological analysis confirmed the known manifestations of Yq deletion, including increased acrosomal curvature and shortening of the apical hook (Figures 2A and 2B). Additionally, both Yq-deleted sperm and shSLY sperm showed a marked reduction in all linear dimension measures and in overall cross-sectional area relative to wild-type sperm, which has not been previously described (Figure 2C). When comparing X- and Y-bearing sperm, for the majority of parameters tested, whether size dependent (linear dimensions and area) or size independent (circularity and regularity), Y-bearing sperm in both mutant genotypes were significantly different from X-bearing sperm and deviated more from wild-type values. A focused analysis of acrosomal curvature and sperm head area (Figure S2) showed that, for both parameters, Y-bearing sperm were more severely affected than X-bearing sperm in the mutant genotypes.

Cluster Analysis Reveals an X/Y Gradient of Morphological Abnormalities

Following this morphometric analysis of sperm dimensions, we carried out dimensional reduction on size-independent angle profiles using t-SNE and used hierarchical clustering to identify different sperm head shape groups within each genetic background. On the MF1 strain background, this separated sperm shapes into two major clusters, the first containing predominantly, but not exclusively, XY^{RIII} sperm and the second containing predominantly, but not exclusively, XY^{RIII}Yqdel sperm. These could

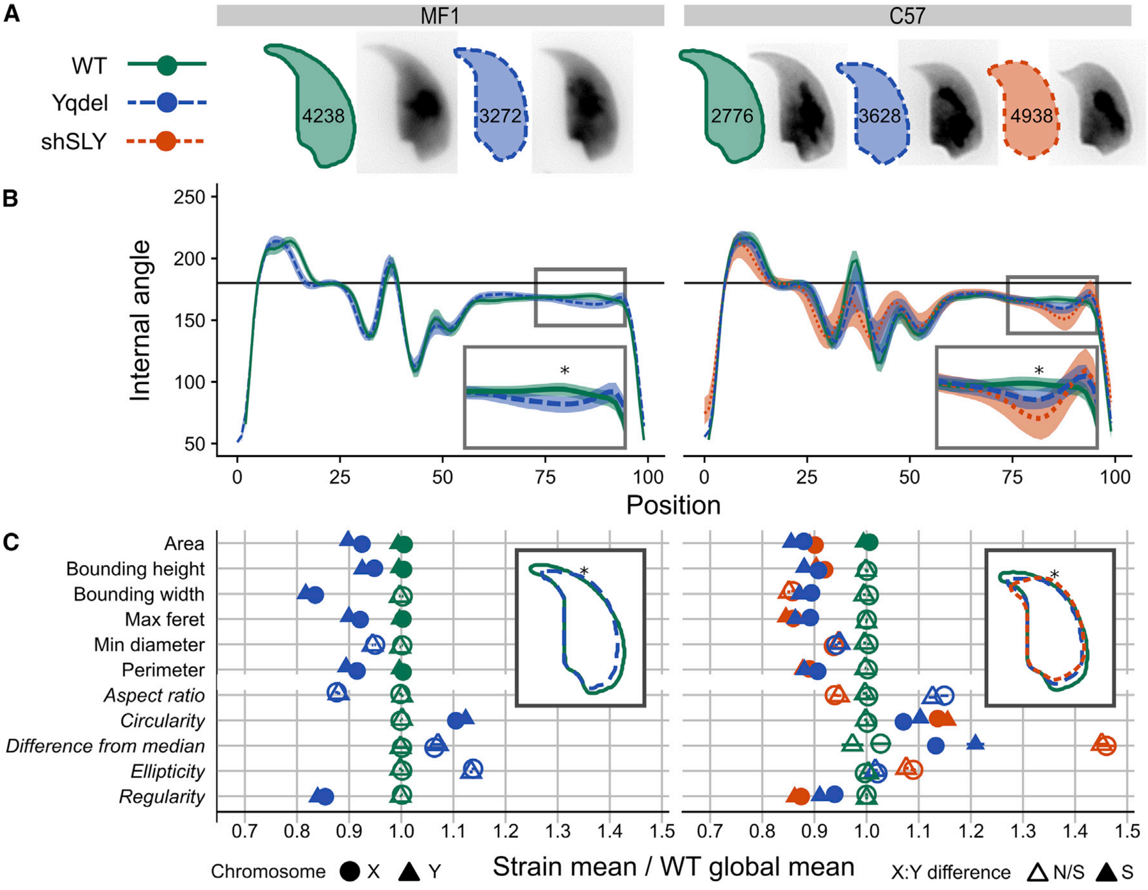

Figure 2. Morphological Differences between Strains

(A) Consensus nuclear outlines for each strain alongside an example DAPI-stained nucleus. Numbers indicate the number of nuclei analyzed for each strain.

(B) Angle profiles from each strain [25]. The x axis is an index representing percentage of the total perimeter as measured counterclockwise from the apex of the sperm hook. The y axis represents the interior angle measured across a sliding window centered on each index location—a smaller angle represents sharper curvature at any given point; thus (e.g.), the hook apex at index 0 shows the smallest angle. The insets highlight the increased acrosomal curvature seen in the mutant genotypes, with "*" indicating the point of greatest difference in curvature between genotypes at index 85.

(C) Comparison of X- and Y-bearing sperm in standard morphometric parameters (see [25] for definitions), compared to mean wild-type values. Italics indicate size-independent parameters. See Table S3 for more details. Inset: overlapping consensus nuclear outlines for each genotype are shown, with the location of index 85 marked *.

See also Figures S2 and S3.

then be subdivided into grades of abnormality, i.e., clusters N1/N2 and A1/A2 (Figures 3A and 3C; Table S2). On the C57Bl6 strain background, the picture was more complex. Once again, there was a cluster of normal-shaped sperm and a continuous spectrum of increasing shape abnormality (clusters A1/A2/A3) corresponding to increasing severity of the Yq gene deficiency phenotype of acrosomal curvature, hook shortening, and area reduction. However, the C57BL6 dataset additionally presented a second axis of shape abnormalities (clusters B1/B2) that encompassed shortening of the sperm nucleus, "flaring" of the base of the sperm head, and exaggeration of the dorsal angle. Because this second type of abnormality was also present at comparable levels in the wild-type (WT) controls, we interpret these as being a strain characteristic unrelated to the Yq gene deficiency phenotype. Finally, a third type of abnormality (cluster S), which encompassed narrowing of the base of the sperm head, almost total loss of acrosomal hook, and effacement of the tail attachment site, was almost exclusive to shSLY sperm (Figures 3B and 3D; Table S2).

On both genetic backgrounds, the wild-type sperm showed an even sex ratio across different shape categories, and the normal-shaped sperm from the mutant genotypes—i.e., cells in categories N1/N2 for MF1 background and N for C57Bl6 background—were preferentially X-bearing sperm; MF1 background: WT X% = 51.3 ± 1.1, Yqdel X% = 59.1 ± 4.6; C57BL6 background: WT X% = 50.2 ± 1.1, Yqdel X% = 58.0 ± 4.7, shSLY X% = 66.7 ± 27.2. Therefore, despite the confounding effects of unrelated background sperm morphology distortions, the cluster analysis overall supports the circularity and regularity measurements showing that, on both genetic backgrounds, Yq deletion and shSLY knockdown lead to more severe morphological defects in Y-bearing sperm. On the C57Bl6 background, we also analyzed sex chromosome localization within the nucleus using dynamic warping [30] (Figure S3). This showed no change

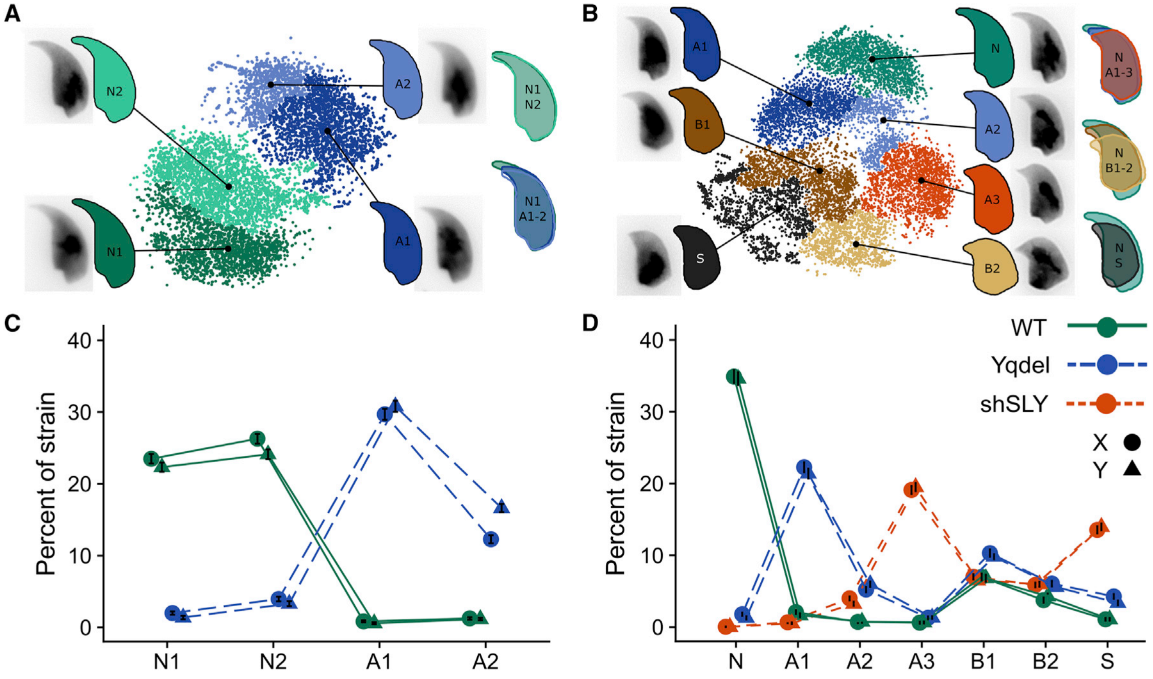

Figure 3. Clustered Sperm Shapes Reveal Morphological Groups

(A and B) Clustered t-SNE plots of angle profiles from MF1 (A) and C57 (B) samples allow groups of nuclei with similar shapes to be distinguished. Consensus nuclear outlines are shown for each cluster alongside a representative DAPI-stained nucleus. Overlapping consensus nuclei allow comparison of clusters to the most "normal" category.

(C and D) The proportion of sperm in each cluster for MF1 (C) and C57 (D) strains is shown, separated into X- and Y-bearing groups. Error bars show the SE of proportion. More clusters with severe abnormalities are enriched for Y-bearing sperm in both backgrounds (see also Tables S2 and S3); C57Bl6 genetic background has additional shape abnormalities (B1 and B2) that are also present in wild-type males and thus are not related to the Yq deficiency phenotype.

in chromosome territory position, indicating that the morphological changes are likely driven by changes in cytoskeletal dynamics during spermiogenesis rather than differences in chromatin organization.

Y-Bearing Sperm Have Lower Motility Than X-Bearing Sperm in XY^{RIII}qdel Males

Because the sex ratio skew is abolished by IVF, the morphological differences alone cannot be the full explanation for the sex ratio skew—i.e., most or all shapes of sperm must be competent to fertilize the oocyte in an IVF context, consistent with prior findings via ICSI in BALB/c wild-type males [31] and in *Sly* knockdown males [6]. Rather, the morphological distortion must impair the sperm's ability to transit *in vivo* from the site of deposition to the fertilization site.

Because sperm selection at the uterotubular junction is largely on the basis of progressive motility [32], we therefore directly tested whether there are motility differences between X- and Y-bearing sperm in XY^{RIII}qdel males, using MF1 strain background animals from our Kent colony. We used a swim-up protocol to separate sperm into six fractions according to their relative motility (STAR Methods). MitoTracker staining confirmed the swim-up fractionation enriched for sperm with longer midpieces in both XY^{RIII} and XY^{RIII}qdel genotypes (Figure S4). This showed a significant enrichment for X-bearing sperm in the upper (more motile) fractions that was not seen in either the wild-type control or the killed sperm control (Figure 4), indicating that Y-bearing sperm from Yqdel males have impaired motility relative to X-bearing sperm.

DISCUSSION

The question of whether there are any morphological or physiological differences between X- and Y-bearing sperm other than their DNA content has provoked controversy for decades [33–37]. Many potential differences have been proposed but none validated, and accumulating understanding of syncytial sperm development has cast doubt on whether such differences are possible even in principle [38–40]. Here, we show that, in Yq-deleted males with offspring sex ratio skewing, the skew is evoked specifically during natural mating/fertilization, is abolished by IVF, is not modified by the epididymal transit time of the sperm, and is not affected by the maternal genetic backgrounds tested. We conclude that the physiological mechanism of the skew cannot be related to any of cumulus penetration, zona pellucida or oolemma binding, sperm/egg fusion, or subsequent embryonic development but must relate to the transport of the sperm to the site of fertilization.

Consistent with this, we show that the morphological abnormalities induced by Yq deletion and/or shSLY knockdown are more severe in Y-bearing sperm than in X-bearing sperm on both genetic backgrounds tested and that Y-bearing sperm from Yqdel males have relatively worse motility than X-bearing sperm on an MF1 background. Because the degree of X sperm

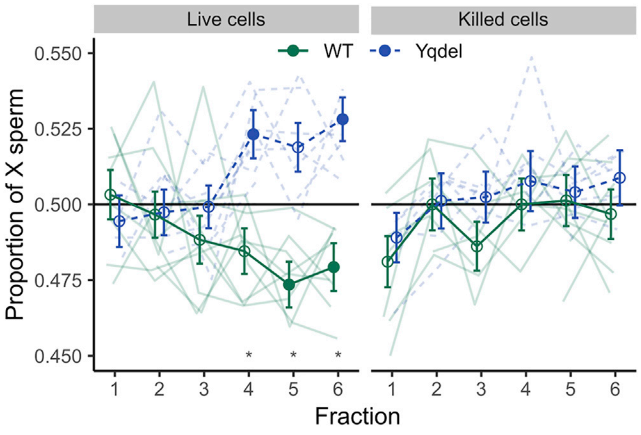

Figure 4. Motility Testing Shows Y-Bearing Sperm Are Poorer Swimmers Than X-Bearing in Yq-del Males

The x axis indicates sperm fractions with relatively increasing motility. Highly motile sperm are enriched for X-bearing over Y-bearing sperm in Yqdel samples but the reverse in wild-type samples. The negative control (freeze-/thaw-killed sperm) showed no enrichment for X or Y sperm in any fraction. Individual samples are shown in faded lines. Stronger lines show the means and SEs of proportion per fraction per genotype. Closed symbols indicate fractions that were individually different from a 50:50 ratio; open symbols indicate fractions that are not significantly different from 50:50 (one sample Z-test; $p < 0.05$ after Bonferroni-Holm multiple testing correction). * indicates fractions where the difference between Yqdel and WT genotypes is significant (two sample Z-test; $p < 0.05$ after Bonferroni-Holm multiple testing correction). Full data on regression parameters are given in Table S4. See also Figure S4.

enrichment we obtained in our *in vitro* swim-up experiment was significantly less pronounced than the sex ratio difference seen *in vivo* during natural mating, it is likely that selection of the most highly motile sperm is more efficient in the environment of the female reproductive tract. We cannot however rule out the possibility that the female tract may also directly select X- versus Y-bearing sperm via (e.g.) surface antigenic differences. We also cannot as yet say whether the changes in sperm shape lead directly to the altered motility by affecting hydrodynamic efficiency or whether the sperm also have altered biomechanics and/or biochemistry (e.g., differential flagellar beat patterns as in the case of *t* heterozygotes [41], differential mitochondrial protein expression as seen in bull sperm [42], and differential capacitation or hyperactivation kinetics). Studying these aspects of the phenotype will require improved methods to separate sperm with differing motility and probe their biochemistry and chromosomal content, with recent advances in microfluidics providing a potential route forward [32].

A final challenge is to elucidate the causal chain between *Slx*/*Sly* genomic competition and sex skewing. These genes interact both with sex chromatin and with histone-modifying enzymes [9, 10] and with the Y-linked histone "reader" protein *Ssty* [5, 43, 44]. This in turn alters postmeiotic sex chromatin and thus coordinately regulates transcription of multiple sex-linked genes in spermatids. However, it is not currently possible to determine which of the hundreds of sex-linked genes regulated by *Slx*/*Sly* are causative for transmission skewing. The results presented here will allow us to prioritize various categories of de-regulated genes in Yqdel animals for functional studies—e.g., by ruling out genes involved in sperm/egg recognition and focusing on genes contributing to sperm motility. A further theoretical clue is given by the only two other examples of male-mediated transmission ratio distortion in mice. Both of these involve at least one "responder" gene whose products escape sharing across the cytoplasmic bridges between syncytially developing spermatids: *Spam1* in the case of the Rb fusions [23–25] and *Smok*^{TCR} in the case of the *t* complex [45, 46]. An alternative is that there is no "responder" gene and that a gene on the X chromosome directly targets a Y-specific DNA element, as in the case of HP1D2 in *Drosophila* [47]. Because *Slx* and *Sly* act as a "thermostat" that can generate both male and female skews depending on the balance of X- and Y-linked amplicons [5], this is hard to reconcile with a simple X-borne, DNA-directed toxin system. We therefore predict that there is a "responder" gene on the X or Y chromosomes that escapes sharing between syncytial spermatids. Recent work indicates that X-linked Toll-like receptors TLR7/8 appear to escape sharing; however, TLR7/8 activation leads to impaired X-bearing sperm function and a reduced number of female offspring [48], opposite to that seen in our model system. Thus, the identity of the responder in Yqdel-mediated distortion remains as yet undetermined.

STAR★METHODS

Detailed methods are provided in the online version of this paper and include the following:

- KEY RESOURCES TABLE
- LEAD CONTACT AND MATERIALS AVAILABILITY
- EXPERIMENTAL MODEL AND SUBJECT DETAILS
  - Mice
- METHOD DETAILS
  - Superovulation and IVF
  - Embryo recovery
  - Timed mating
  - Sperm collection and fixation
  - Test tube swim-up motility fractionation
  - Image capturing and FISH of sperm
- QUANTIFICATION AND STATISTICAL ANALYSIS
  - Sperm morphological analysis
  - Sperm motility analysis
- DATA AND CODE AVAILABILITY

SUPPLEMENTAL INFORMATION

Supplemental Information can be found online at https://doi.org/10.1016/j.cub.2019.09.031.

ACKNOWLEDGMENTS

We thank the animal handling staff at the University of Kent, University of Cambridge, Cochin Institute, and Charles River Laboratories. B.M.S. was supported by the Biotechnology and Biological Sciences Research Council (BBSRC) (BB/N000129/1). P.J.I.E. and C.C.R. were supported by H.E.F.C.E. (University of Kent) and by the BBSRC (BB/N000463/1). E.E.P.J. was supported by BBSRC training grant BB/L502443/1 and Genus PLC. J.C. and C.I.-R. were supported by INSERM. Their research was funded by ANR (Agence Nationale pour la Recherche) (ANR-12–JSV2-0005–01 and ANR-17-CE12-0004-01 to J.C.).

AUTHOR CONTRIBUTIONS

Conceptualization, P.J.I.E.; Methodology, B.M.S. and P.J.I.E.; Software and Validation, B.M.S., C.C.R., and P.J.I.E.; Investigation, C.C.R., B.M.S., E.E.P.J., D.D., C.P., G.S., and C.I.-R.; Data Curation and Formal Analysis, C.C.R., B.M.S., and P.J.I.E.; Visualization, B.M.S. and P.J.I.E.; Supervision and Project Administration, P.J.I.E., N.A.A., and J.C.; Writing – Original Draft, P.J.I.E., B.M.S., and C.C.R.; Writing – Review & Editing, P.J.I.E., B.M.S., C.C.R., and G.S.; Resources, J.C.; Funding Acquisition, P.J.I.E., N.A.A., and J.C. All authors gave final approval for publication.

DECLARATION OF INTERESTS

The authors declare no competing interests.

Received: May 24, 2019
Revised: August 2, 2019
Accepted: September 12, 2019
Published: October 17, 2019

REFERENCES

1. Mueller, J.L., Mahadevaiah, S.K., Park, P.J., Warburton, P.E., Page, D.C., and Turner, J.M.A. (2008). The mouse X chromosome is enriched for multicopy testis genes showing postmeiotic expression. Nat. Genet. *40*, 794–799.
2. Soh, Y.Q.S., Alföldi, J., Pyntikova, T., Brown, L.G., Graves, T., Minx, P.J., Fulton, R.S., Kremitzki, C., Koutseva, N., Mueller, J.L., et al. (2014). Sequencing the mouse Y chromosome reveals convergent gene acquisition and amplification on both sex chromosomes. Cell *159*, 800–813.
3. Cocquet, J., Ellis, P.J.I., Yamauchi, Y., Mahadevaiah, S.K., Affara, N.A., Ward, M.A., and Burgoyne, P.S. (2009). The multicopy gene Sly represses the sex chromosomes in the male mouse germline after meiosis. PLoS Biol. *7*, e1000244.
4. Cocquet, J., Ellis, P.J.I., Yamauchi, Y., Riel, J.M., Karacs, T.P.S., Rattigan, A., Ojarikre, O.A., Affara, N.A., Ward, M.A., and Burgoyne, P.S. (2010). Deficiency in the multicopy Sycp3-like X-linked genes Slx and Slxl1 causes major defects in spermatid differentiation. Mol. Biol. Cell *21*, 3497–3505.
5. Cocquet, J., Ellis, P.J.I., Mahadevaiah, S.K., Affara, N.A., Vaiman, D., and Burgoyne, P.S. (2012). A genetic basis for a postmeiotic X versus Y chromosome intragenomic conflict in the mouse. PLoS Genet. *8*, e1002900.
6. Riel, J.M., Yamauchi, Y., Sugawara, A., Li, H.Y.J., Ruthig, V., Stoytcheva, Z., Ellis, P.J.I., Cocquet, J., and Ward, M.A. (2013). Deficiency of the multi-copy mouse Y gene Sly causes sperm DNA damage and abnormal chromatin packaging. J. Cell Sci. *126*, 803–813.
7. Bachtrog, D. (2014). Signs of genomic battles in mouse sex chromosomes. Cell *159*, 716–718.
8. Reynard, L.N., Turner, J.M.A., Cocquet, J., Mahadevaiah, S.K., Touré, A., Höög, C., and Burgoyne, P.S. (2007). Expression analysis of the mouse multi-copy X-linked gene Xlr-related, meiosis-regulated (Xmr), reveals that Xmr encodes a spermatid-expressed cytoplasmic protein, SLX/XMR. Biol. Reprod. *77*, 329–335.
9. Reynard, L.N., Cocquet, J., and Burgoyne, P.S. (2009). The multi-copy mouse gene Sycp3-like Y-linked (Sly) encodes an abundant spermatid protein that interacts with a histone acetyltransferase and an acrosomal protein. Biol. Reprod. *81*, 250–257.
10. Zhuang, X.J., Hou, X.J., Liao, S.-Y., Wang, X.-X., Cooke, H.J., Zhang, M., and Han, C. (2011). SLXL1, a novel acrosomal protein, interacts with DKKL1 and is involved in fertilization in mice. PLoS ONE *6*, e20866.
11. Moretti, C., Serrentino, M.-E., Ialy-Radio, C., Delessard, M., Soboleva, T.A., Tores, F., Leduc, M., Nitschké, P., Drevet, J.R., Tremethick, D.J., et al. (2017). SLY regulates genes involved in chromatin remodeling and interacts with TBL1XR1 during sperm differentiation. Cell Death Differ. *24*, 1029–1044.
12. Moriwaki, K. (1988). Genetic factors governing sperm morphology of the mouse. Mouse News Lett. *82*, 138.
13. Conway, S.J., Mahadevaiah, S.K., Darling, S.M., Capel, B., Rattigan, A.M., and Burgoyne, P.S. (1994). Y353/B: a candidate multiple-copy spermiogenesis gene on the mouse Y chromosome. Mamm. Genome *5*, 203–210.
14. Touré, A., Szot, M., Mahadevaiah, S.K., Rattigan, A., Ojarikre, O.A., and Burgoyne, P.S. (2004). A new deletion of the mouse Y chromosome long arm associated with the loss of Ssty expression, abnormal sperm development and sterility. Genetics *166*, 901–912.
15. Ellis, P.J.I., Clemente, E.J., Ball, P., Touré, A., Ferguson, L., Turner, J.M.A., Loveland, K.L., Affara, N.A., and Burgoyne, P.S. (2005). Deletions on mouse Yq lead to upregulation of multiple X- and Y-linked transcripts in spermatids. Hum. Mol. Genet. *14*, 2705–2715.
16. MacBride, M.M., Navis, A., Dasari, A., and Perez, A.V. (2017). Mild reproductive impact of a Y chromosome deletion on a C57BL/6J substrain. Mamm. Genome *28*, 155–165.
17. Larson, E.L., Kopania, E.E.K., and Good, J.M. (2018). Spermatogenesis and the evolution of mammalian sex chromosomes. Trends Genet. *34*, 722–732.
18. Sangrithi, M.N., and Turner, J.M.A. (2018). Mammalian X chromosome dosage compensation: perspectives from the germ line. BioEssays *40*, e1800024.
19. Hughes, J.F., and Page, D.C. (2015). The biology and evolution of mammalian Y chromosomes. Annu. Rev. Genet. *49*, 507–527.
20. van der Heijden, G.W., Eijpe, M., and Baarends, W.M. (2011). The X and Y chromosome in meiosis: how and why they keep silent. Asian J. Androl. *13*, 779–780.
21. Ward, M.A., and Burgoyne, P.S. (2006). The effects of deletions of the mouse Y chromosome long arm on sperm function–intracytoplasmic sperm injection (ICSI)-based analysis. Biol. Reprod. *74*, 652–658.
22. Aranha, I.P., and Martin-DeLeon, P.A. (1995). Mouse chromosome 6 in Rb translocations: consequences in singly and doubly heterozygous males. Cytogenet. Cell Genet. *69*, 253–259.
23. Zheng, Y., Deng, X., and Martin-DeLeon, P.A. (2001). Lack of sharing of Spam1 (Ph-20) among mouse spermatids and transmission ratio distortion. Biol. Reprod. *64*, 1730–1738.
24. Zheng, Y., Deng, X., Zhao, Y., Zhang, H., and Martin-DeLeon, P.A. (2001). Spam1 (PH-20) mutations and sperm dysfunction in mice with the Rb(6.16) or Rb(6.15) translocation. Mamm. Genome *12*, 822–829.
25. Martin-DeLeon, P.A., Zhang, H., Morales, C.R., Zhao, Y., Rulon, M., Barnoski, B.L., Chen, H., and Galileo, D.S. (2005). Spam1-associated transmission ratio distortion in mice: elucidating the mechanism. Reprod. Biol. Endocrinol. *3*, 32.
26. Hadjantonakis, A.-K., Gertsenstein, M., Ikawa, M., Okabe, M., and Nagy, A. (1998). Generating green fluorescent mice by germline transmission of green fluorescent ES cells. Mech. Dev. *76*, 79–90.
27. Kotarska, K., Galas, J., Przybyło, M., Bilińska, B., and Styrna, J. (2015). Increased progesterone production in cumulus-oocyte complexes of female mice sired by males with the Y-chromosome long arm deletion and its potential influence on fertilization efficiency. Reprod. Sci. *22*, 242–249.
28. Kotarska, K., and Lenartowicz, M. (2011). Sperm migration and selection in the reproductive tract of female mice is mostly affected by male genotype. Folia Biol. (Krakow) *59*, 71–75.
29. Skinner, B.M., Rathje, C.C., Bacon, J., Johnson, E.E.P., Larson, E.L., Kopania, E.E.K., Good, J.M., Yousafzai, G., Affara, N.A., and Ellis, P.J.I. (2019). A high-throughput method for unbiased quantitation and categorization of nuclear morphology. Biol. Reprod. *100*, 1250–1260.
30. Skinner, B.M., Bacon, J., Rathje, C.C., Larson, E.L., Kopania, E.E.K., Good, J.M., Affara, N.A., and Ellis, P.J.I. (2019). Automated nuclear cartography reveals conserved sperm chromosome territory localization across 2 million years of mouse evolution. Genes (Basel) *10*, 109.

31. Burruel, V.R., Yanagimachi, R., and Whitten, W.K. (1996). Normal mice develop from oocytes injected with spermatozoa with grossly misshapen heads. Biol. Reprod. *55*, 709–714.

32. Zaferani, M., Palermo, G.D., and Abbaspourrad, A. (2019). Strictures of a microchannel impose fierce competition to select for highly motile sperm. Sci. Adv. *5*, v2111.

33. Carvalho, J.O., Silva, L.P., Sartori, R., and Dode, M.A.N. (2013). Nanoscale differences in the shape and size of X and Y chromosome-bearing bovine sperm heads assessed by atomic force microscopy. PLoS ONE *8*, e59387.

34. Cui, K.H. (1997). Size differences between human X and Y spermatozoa and prefertilization diagnosis. Mol. Hum. Reprod. *3*, 61–67.

35. Grant, V.J. (2006). Entrenched misinformation about X and Y sperm. BMJ *332*, 916.

36. van Munster, E.B., Stap, J., Hoebe, R.A., te Meerman, G.J., and Aten, J.A. (1999). Difference in sperm head volume as a theoretical basis for sorting X- and Y-bearing spermatozoa: potentials and limitations. Theriogenology *52*, 1281–1293.

37. Révay, T., Nagy, S., Kovács, A., Edvi, M.E., Hidas, A., Rens, W., and Gustavsson, I. (2004). Head area measurements of dead, live, X- and Y-bearing bovine spermatozoa. Reprod. Fertil. Dev. *16*, 681–687.

38. Braun, R.E., Behringer, R.R., Peschon, J.J., Brinster, R.L., and Palmiter, R.D. (1989). Genetically haploid spermatids are phenotypically diploid. Nature *337*, 373–376.

39. Caldwell, K.A., and Handel, M.A. (1991). Protamine transcript sharing among postmeiotic spermatids. Proc. Natl. Acad. Sci. USA *88*, 2407–2411.

40. Ellis, P.J.I., Yu, Y., and Zhang, S. (2011). Transcriptional dynamics of the sex chromosomes and the search for offspring sex-specific antigens in sperm. Reproduction *142*, 609–619.

41. Katz, D.F., Erickson, R.P., and Nathanson, M. (1979). Beat frequency is bimodally distributed in spermatozoa from T/t12 mice. J. Exp. Zool. *210*, 529–535.

42. Scott, C., de Souza, F.F., Aristizabal, V.H.V., Hethrington, L., Krisp, C., Molloy, M., Baker, M.A., and Dell'Aqua, J.A. (2018). Proteomic profile of sex-sorted bull sperm evaluated by SWATH-MS analysis. Anim. Reprod. Sci. *198*, 121–128.

43. Eberl, H.C., Spruijt, C.G., Kelstrup, C.D., Vermeulen, M., and Mann, M. (2013). A map of general and specialized chromatin readers in mouse tissues generated by label-free interaction proteomics. Mol. Cell *49*, 368–378.

44. Comptour, A., Moretti, C., Serrentino, M.-E., Auer, J., Ialy-Radio, C., Ward, M.A., Touré, A., Vaiman, D., and Cocquet, J. (2014). SSTY proteins co-localize with the post-meiotic sex chromatin and interact with regulators of its expression. FEBS J. *281*, 1571–1584.

45. Herrmann, B.G., Koschorz, B., Wertz, K., McLaughlin, K.J., and Kispert, A. (1999). A protein kinase encoded by the t complex responder gene causes non-mendelian inheritance. Nature *402*, 141–146.

46. Véron, N., Bauer, H., Weisse, A.Y., Lüder, G., Werber, M., and Herrmann, B.G. (2009). Retention of gene products in syncytial spermatids promotes non-Mendelian inheritance as revealed by the t complex responder. Genes Dev. *23*, 2705–2710.

47. Helleu, Q., Gérard, P.R., Dubruille, R., Ogereau, D., Prud'homme, B., Loppin, B., and Montchamp-Moreau, C. (2016). Rapid evolution of a Y-chromosome heterochromatin protein underlies sex chromosome meiotic drive. Proc. Natl. Acad. Sci. USA *113*, 4110–4115.

48. Umehara, T., Tsujita, N., and Shimada, M. (2019). Activation of Toll-like receptor 7/8 encoded by the X chromosome alters sperm motility and provides a novel simple technology for sexing sperm. PLoS Biol. *17*, e3000398.

49. Krzanowska, H. (1981). Sperm head abnormalities in relation to the age and strain of mice. J. Reprod. Fertil. *62*, 385–392.

50. Lawitts, J.A., and Biggers, J.D. (1993). Culture of preimplantation embryos. Methods Enzymol. *225*, 153–164.

51. Schneider, C.A., Rasband, W.S., and Eliceiri, K.W. (2012). NIH Image to ImageJ: 25 years of image analysis. Nat. Methods *9*, 671–675.

52. Prieto, G., Chevalier, M., and Guibelalde, E. (2009). MS_SSIM Index: a Java plugin in ImageJ. https://webs.ucm.es/info/fismed/MSSIM/MSSIM_archivos/frame.htm.

53. Quinn, P., Kerin, J.F., and Warnes, G.M. (1985). Improved pregnancy rate in human in vitro fertilization with the use of a medium based on the composition of human tubal fluid. Fertil. Steril. *44*, 493–498.

54. R Core Team (2018). R: A language and environment for statistical computing (R Foundation for Statistical Computing). https://www.R-project.org.

55. van der Maaten, L. (2014). Accelerating t-SNE using tree-based algorithms. J. Mach. Learn. Res. *15*, 3221–3245.

56. Krijthe, J. (2019). R wrapper for Van der Maaten's Barnes-Hut implementation of t-distributed stochastic neighbor embedding. https://github.com/jkrijthe/Rtsne.

57. Kaufman, L., and Rousseeuw, P.J. (1990). Finding Groups in Data: An Introduction to Cluster Analysis (John Wiley & Sons).

58. Maechler, M., Rousseeuw, P., Struyf, A., Hubert, M., Hornik, K., Studer, M., Roudier, P., Gonzalez, J., Kozlowski, K., Schubert, E., et al. (2019). cluster: "finding groups in data": cluster analysis extended Rousseeuw et al. https://CRAN.R-project.org/package=cluster.

59. Cribari-Neto, F., and Zeileis, A. (2010). Beta regression in R. J. Stat. Softw. *34*, 1–24.

60. Mangiafico, S. (2019). rcompanion: functions to support extension education program evaluation. https://CRAN.R-project.org/package=rcompanion.

STAR★METHODS

KEY RESOURCES TABLE

REAGENT or RESOURCE	SOURCE	IDENTIFIER
Chemicals, Peptides, and Recombinant Proteins		
PMSG	NVS	Cat#859448
hCG	NVS	Cat#804745
HTF media	[43]	N/A
KSOM media	[44]	N/A
Hyaluronidase	Sigma	Cat#H4272
Mouse chromosome X paint	Cytocell	Cat#AMP-0XG
Mouse chromosome Y paint	Cytocell	Cat#AMP-0YR
MitoTracker Red	Life Technologies	Cat#M7512
Experimental Models: Organisms/Strains		
Mouse: MF1-XY^{RIII}	Colonies were sourced from Dr Paul Burgoyne, NIMR.	[13]
Mouse: MF1-$X^{GFP}Y^{RIII}$	Colonies were sourced from Dr Paul Burgoyne, NIMR.	[22]
Mouse: MF1-XY^{RIII}qdel	Colonies were sourced from Dr Paul Burgoyne, NIMR.	[13]
Mouse: MF1-$X^{GFP}Y^{RIII}$qdel	This study (F1 cross of above two lines)	N/A
Mouse: C57Bl6/N-XY^{RIII}	Colonies were sourced from Dr Paul Burgoyne, NIMR, and backcrossed onto C57Bl6/N background	[13]
Mouse: C57Bl6/N-XY^{RIII}qdel	Colonies were sourced from Dr Paul Burgoyne, NIMR, and backcrossed onto C57Bl6/N background.	[13]
Mouse: C57Bl6/N-XY^{RIII}-shSLY	Colonies were sourced from Dr Paul Burgoyne, NIMR, and backcrossed onto C57Bl6/N background	[3]
Mouse: MF1 (females used to maintain breeding colonies in Cambridge and Kent, and as oocyte donors for some IVF experiments)	Sourced from Charles River Laboratories	(discontinued strain)
Mouse: B6CBA/F1 (females used as oocyte donors for some IVF experiments)	Sourced from Charles River Laboratories	https://www.criver.com/products-services/find-model/B6CBAf1-mouse
Mouse: C57BL/6NRj (background strain for breeding colonies in France)	Sourced from Janvier Labs	https://www.janvier-labs.com/en/fiche_produit/c57bl-6nrj_mouse/
Software and Algorithms		
Nuclear Morphology Analysis 1.15.1	[25]	https://bitbucket.org/bmskinner/nuclear_morphology/wiki/Home
ImageJ	[45]	https://imagej.nih.gov/ij/
R 3.5.1	[47]	https://www.r-project.org/
Rtsne v0.15	[49]	CRAN (https://cran.r-project.org/)
cluster v2.0.7-1	[50]	CRAN (https://cran.r-project.org/)
betareg *v3.1.1*	[51]	CRAN (https://cran.r-project.org/)
rcompanion v2.1.1	[52]	CRAN (https://cran.r-project.org/)
Other		
Nuclear morphological measurements and analysis scripts	This paper	https://github.com/bmskinner/Yqdel_physiology

LEAD CONTACT AND MATERIALS AVAILABILITY

Further information and requests for resources and reagents should be directed to and will be fulfilled by the Lead Contact, Peter Ellis (P.J.I.Ellis@kent.ac.uk). The mouse strains used were originally obtained from colonies developed by Dr Paul Burgoyne (NIMR, Mill Hill, London) under an MTA that precludes further transfer - requests for these strains should be directed to the Francis Crick Institute, London as current owners of the strains.

EXPERIMENTAL MODEL AND SUBJECT DETAILS

Mice

All animal procedures were in accordance with the United Kingdom Animal Scientific Procedures Act 1986 and were subject to local ethical review in UK and France (*Comite d'Ethique pour l'Experimentation Animale*, *Universite Paris Descartes*). Animals on MF1 strain background were bred on Home Office licenses 80/2451 and 70/8925, held by PE. These strains were originally sourced from colonies developed by Dr Paul Burgoyne (NIMR, Mill Hill, London), and were subsequently bred at Cambridge University Central Biomedical Services, or on contract by Charles River Laboratories (Manston, Kent, UK). Animals on C57Bl6/N background were bred at the Cochin Institute animal facility (license held by JC, registration number CEEA34.JC.114.12). These strains were originally sourced from colonies developed by Dr Paul Burgoyne (NIMR, Mill Hill, London) and were subsequently backcrossed to C57Bl6/N animals at the Cochin Institute animal facility (to reach > 95% of C57Bl6/N background). On both backgrounds, all males studied carry an RIII-derived Y chromosome. This provides the appropriate comparison for the $^2/_3$ Yq deletion, which arose on a RIII background. Unless specified otherwise, breeding animals were housed as pairs or trios, and progeny housed singly or in small groups (< 4 per cage), with *ad libitum* access to food and water throughout and a 12/12 hour light/dark cycle. Animals bred at Cambridge were housed in individually ventilated cages, animals bred at Kent or in France were housed in standard cages within a barrier facility. Animals used in this study were sacrificed via CO_2 followed by cervical dislocation (motility experiments) or cervical dislocation only (morphology experiments) and tissues collected post mortem for analysis. Full details of the animals used in this study are given in Table S3. All animals were older than 10 weeks, after which the testes produce mature morphologically normal sperm [49].

METHOD DETAILS

Unless otherwise stated, room temperature (RT) was approximately 25°C.

Superovulation and IVF

For the IVF work, and for *in vivo* experiments requiring superovulation, 5-6 week old females were induced to superovulate with injections of 5 IU PMSG (NVS Cat no. 859448) and 5 IU hCG (NVS Cat no.804745) in 100-150 μL of PBS, given 48 h apart. Donor males were sacrificed by cervical dislocation. One cauda epididymis was dissected and transferred to a 250 μL pre-equilibrated (37°C, 5% CO_2) droplet of HTF media [53] supplemented with 5% BSA, under embryo safe mineral oil (Sigma). The cauda was opened with a scalpel and the sperm allowed to swim out and capacitate for 1 hr at 37°C, 5% CO_2. Next, superovulated females (4 for each cumulus-on and cumulus-off experimental sample) were sacrificed. Cumulus/oocyte complexes were retrieved from the ampulla of the oviduct and transferred either into a 500 μL pre-equilibrated droplet of either HTF media or HTF media with hyaluronidase (300 μg/ml, Sigma H4272) and incubated for 3 minutes. The decumulated oocytes were rinsed briefly through a second HTF droplet and transferred to a final 500 μL droplet of HTF media without hyaluronidase. 3 μL of capacitated sperm were added to each fertilization droplet and incubated for 4 hr to allow fertilization. After fertilization, embryos were rinsed briefly through a fresh HTF droplet and transferred to culture wells containing 1ml KSOM media [50] + 3mg/ml BSA for overnight incubation. The following morning, cleavage-stage embryos were transferred to KSOM media without BSA and cultured to blastocyst stage for GFP scoring. Total fertilization rates were 78.4% for cumulus-on oocytes and 82.5% for decumulated oocytes (not significant). Progression from 2-cell to blastocyst stages was 95.7% for cumulus-on versus 89.8% for cumulus-off groups ($p = 0.013$, binomial test for difference of proportions).

Embryo recovery

For the 2-cell embryo recovery experiment, superovulated females were housed with males overnight (2 per male tested), and checked for plugs the following morning. The day of plug identification was recorded as day 1. Females were sacrificed on the day following plug identification. Two-cell embryos were recovered and cultured to the blastocyst stage to score GFP expression. Experiments were performed using both CBAB6/F1 egg donors (9 $X^{GFP}Y^{RIII}$ males and $X^{GFP}Y^{RIII}$qdel males tested) and MF1 egg donors (4 $X^{GFP}Y^{RIII}$ males and $X^{GFP}Y^{RIII}$qdel males tested).

For the series of experiments with CBAB6/F1 egg donors, each male was mated again to another pair of superovulated CBAB6/F1 females 3 days after the first mating, and embryos collected and scored as above. The sperm for this second mating must have had only a short residence time in the epididymis, i.e., it is "fresher" than the sperm from the original mating. For four $X^{GFP}Y^{RIII}$ males and four $X^{GFP}Y^{RIII}$qdel this cycle was repeated again, with a third mating after a further 2-3 weeks (i.e., fertilization with aged sperm), and then finally a fourth mating 3 days later (i.e., fertilization with fresh sperm). This method of varying the mating interval to ensure that

eggs are fertilized either by fresh sperm (3 day interval since previous mating) or sperm that have undergone aging in the epididymis (virgin mating or > 14 day interval since previous mating) has been previously shown to affect the magnitude of transmission ratio skewing in Rb fusion carriers [22].

We found no effect of mating interval for either of the male genotypes, and the difference in sex ratio (defined as GFP+ve / total number of blastocysts) between CBAB6/F1 and MF1 oocyte donors was also not statistically significant for either of the male genotypes ($p > 0.1$, two-tailed two-proportions Z-test). The results were thus pooled across all experiments when comparing between $X^{GFP}Y^{RIII}$ and $X^{GFP}Y^{RIII}$qdel genotypes. 95%–97% of recovered 2-cell embryos progressed to blastocyst stage (327/329 wild-type, 511/538 Yq-del). The difference in progression between $X^{GFP}Y^{RIII}$ and $X^{GFP}Y^{RIII}$qdel genotypes was non-significant ($p = 0.39$, two-tailed two-proportions Z-test), and also too small in magnitude to explain the sex ratio skew.

Timed mating

For timed mating experiments looking at the effects of female background, estrus cycle progression was staged via visual inspection, females in estrus were housed with males overnight, and checked daily until plugged. Each male was mated successively at 1-week intervals to 4 different females: 2 from the XY^{RIII} colony and two from the XY^{RIII}qdel colony. This allowed replicate data to be collected for both maternal backgrounds for each male tested. Once plugged, females were boxed out, sacrificed mid-gestation (~day 13.5 post *coitus*), and the number of green (XX) versus non-fluorescent (XY) embryos counted. The number of reabsorbing embryos was counted and found to be independent of both male and female genotypes (male: 20/254 XY^{RIII}, 17/302 XY^{RIII}qdel, $p = 0.38$; female: 12/254 XY^{RIII} daughters, 25/302 XY^{RIII}qdel daughters, $p = 0.13$, two-tailed two-proportions Z-tests).

Sperm collection and fixation

Mice were sacrificed by cervical dislocation and sperm collected from cauda epididymis and vas deferens as previously described [29]. The sperm samples were rinsed in 3 washes of 1xPBS and fixed in 3:1 methanol:acetic acid. Table S1 shows details of the sperm samples analyzed in the work reported here. Initial experiments used pooled samples, subsequent experiments analyzed individual males. No differences were observed in summary statistics from pooled versus individual samples (Wilcoxon rank sum tests, $p > 0.05$).

Test tube swim-up motility fractionation

2 caudae epidydimides from freshly dissected males were transferred to 200 μL pre-equilibrated (37°C, 5% CO_2) HTF droplets under embryo safe mineral oil (Sigma), opened with a scalpel and the sperm allowed to swim out and capacitate for 1 hr at 37°C, 5% CO_2. Following capacitation, general motility was checked under a light microscope before carefully transferring 50 μL sperm suspension to the bottom of a test tube (Greiner Bio-One #120160) with 3 mL of pre-equilibrated (37°C, 5% CO_2) HTF media. Sperm were allowed to swim up through the overlying column of media for 30 minutes, and then successive 0.5ml aliquots were carefully pipetted from the top of the liquid meniscus, yielding six successive fractions from different swim depths.

In one experiment (Figure 4, n = 9 XY^{RIII}, 10 XY^{RIII}qdel males, MF1 background), these fractions were immediately spun down onto glass slides, processed for X/Y FISH, and counted blind (i.e., the counter was not aware of which slide contained which fraction or genotype). However, since the best swimming fractions had fewer sperm, and the Yq-del phenotype is readily visible, perfect blinding was not possible.

In another experiment (Figure S3, n = 5 XY^{RIII} and 5 XY^{RIII}qdel males), the top and bottom fractions only were stained using 200 nM MitoTracker Red (M7512, Life Technologies) for 30 minutes at 37°C, 5% CO_2, and then fixed as described above. Sperm were spun down and imaged at lower magnification (x60 objective) to allow visualization of the whole midpiece. 5 males were analyzed per genotype, and 55 images taken per fraction per male, each image containing 1-3 cells. Consistent image exposure times were used to allow accurate relative quantification of fluorescence intensity. Midpiece length and average staining intensity were measured using ImageJ [51].

Image capturing and FISH of sperm

In order to compare X and Y-bearing sperm we performed a capture / recapture analysis in which we first imaged sperm nuclei using DAPI fluorescence microscopy, then subjected them to FISH labeling and re-imaged them to determine their X/Y status. This was necessary because the chromatin swelling step required for FISH probe penetration distorts the detailed morphology of the sperm head (Figure S1).

Fixed sperm were dropped onto poly-L-lysine coated slides, air-dried, stained using Vectorshield Antifade mounting medium with DAPI (Vector Laboratories, Peterborough, UK), and covered with 22x50mm coverslips. Slides were imaged on an Olympus BX61 epifluorescence microscope equipped with cooled CCDs and appropriate filters, and a motorised stage (Prior Scientific, Cambridge, UK). Images were captured using SmartCapture 3 (Digital Scientific, Cambridge, UK) and exported in TIFF format for downstream analyses. XYZ coordinates were recorded for each image taken to allow re-identification of cells. Slides were washed in 2xSSC for 2x10 minutes to remove mounting medium, and dehydrated through an ethanol series (70%, 80%, 100%, 2mins at RT). FISH was performed as previously described [30] using X and Y chromosome paints (Cytocell, Cambridge, UK), and images were captured as described above. The saved slide positions were used to perform automated batch capture of the previously imaged nuclei. Similarities between chromosome positions were assessed using MS-SSIM* [52], as described in [30].

QUANTIFICATION AND STATISTICAL ANALYSIS

Statistical parameters including the statistical tests used, values of n, and statistical significance are reported in the Figure Legends and in this STAR Methods section. Results are expressed as mean ± standard error of the mean (S. E. M.) for directly measured parameters, and as mean ± standard error of proportion (S. E. P.) for proportional measures.

Sperm morphological analysis

Morphology analysis was performed using the ImageJ plugin 'Nuclear Morphology Analysis' [29], version 1.15.1, available at https://bitbucket.org/bmskinner/nuclear_morphology/wiki/Home. In addition to the previously-described measurements, we developed a simple user interface to allow FISH images and pre-FISH images to be displayed side by side. This allowed simple manual sorting of pre-FISH sperm images into groups of X-bearing versus Y-bearing sperm to allow comparison. Data were exported for further processing in R 3.5.1 [54]. Summary statistics were calculated per sample and per strain for each chromosome, with errors propagated by quadrature. Two-dimensional Barnes-Hut tSNE [55] was run on angle profiles using *Rtsne*, version 0.15 [56] (perplexity = 100, max_iter = 1000) and a consistent seed. Parameters were chosen following confirmation of plot consistency across a range of values. Hierarchical clustering was performed by agglomerative nesting [57] using Ward's method via the *cluster* package, version 2.0.7-1 [58]. Ward's method was selected as giving the highest agglomerative coefficient. Dendrograms were cut, with cluster number determined following visual inspection, and cell cluster membership was imported back into Nuclear Morphology Analysis to allow consensus nucleus building. FISH images from males on the C57BL6 background were further used to assess nuclear organization via dynamic signal warping, as previously described [30].

Differences in measured strain parameters between X- and Y-bearing sperm were tested using non-parametric Wilcoxon rank sum tests. Significance was assessed as $p < 0.01$ following Bonferroni multiple testing correction.

Sperm motility analysis

For X/Y counting of swim-up slides, the cell-containing area of the slide was systematically scanned at high magnification (100x objective), and all observed cells scored for X/Y status. Counting was continued until at least 400 cells had been counted for each slide stained, or until all cells on the slide had been counted if fewer than 400 were present in any given fraction. The data were modeled using a beta regression using a logit link, with X proportion explained as an interaction of strain, live/dead state, and segment using the *betareg* R package, version 3.1.1 [59], and likelihood ratio testing was performed using the *rcompanion* package, version 2.1.1 [60].

For comparisons of MitoTracker staining of best-swimming (n = 495 WT, 401 Yq-del) and poorest-swimming fractions (n = 285 WT, 656 Yq-del), differences between fractions within each genotype were assessed using two-sample two-tailed Kolmogorov-Smirnov tests, with significance assessed as $p < 0.01$.

DATA AND CODE AVAILABILITY

Full data for breeding and embryo recovery experiments as well as the measured parameters for X and Y bearing nuclei, and the R code used to perform the analysis are available on Github (https://github.com/bmskinner/Yqdel_physiology).

Current Biology, Volume *29*

Supplemental Information

Differential Sperm Motility Mediates the Sex

Ratio Drive Shaping Mouse Sex Chromosome Evolution

Claudia Cattoni Rathje, Emma Elizabeth Philippa Johnson, Deborah Drage, Christina Patinioti, Giuseppe Silvestri, Nabeel Ahmed Affara, Côme Ialy-Radio, Julie Cocquet, Benjamin Matthew Skinner, and Peter James Ivor Ellis

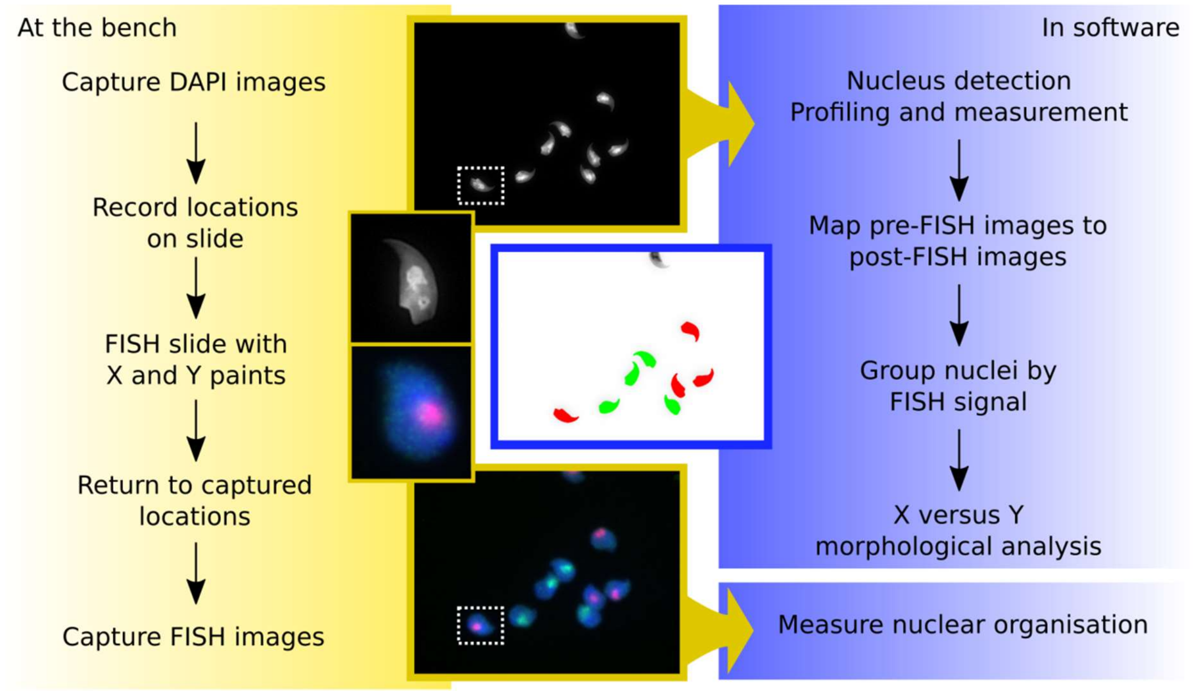

Figure S1. Flowchart showing the capture-recapture protocol. Related STAR Methods. Flowchart showing the capture-recapture protocol. The extensive swelling required for FISH probes to penetrate the nucleus (inset nuclei show before and after FISH) requires storing slide coordinates and imaging the slide before and after FISH for morphology analysis and subsequent XY grouping. A software user interface allows simple assignment of pre-FISH nuclei based on post-FISH images.

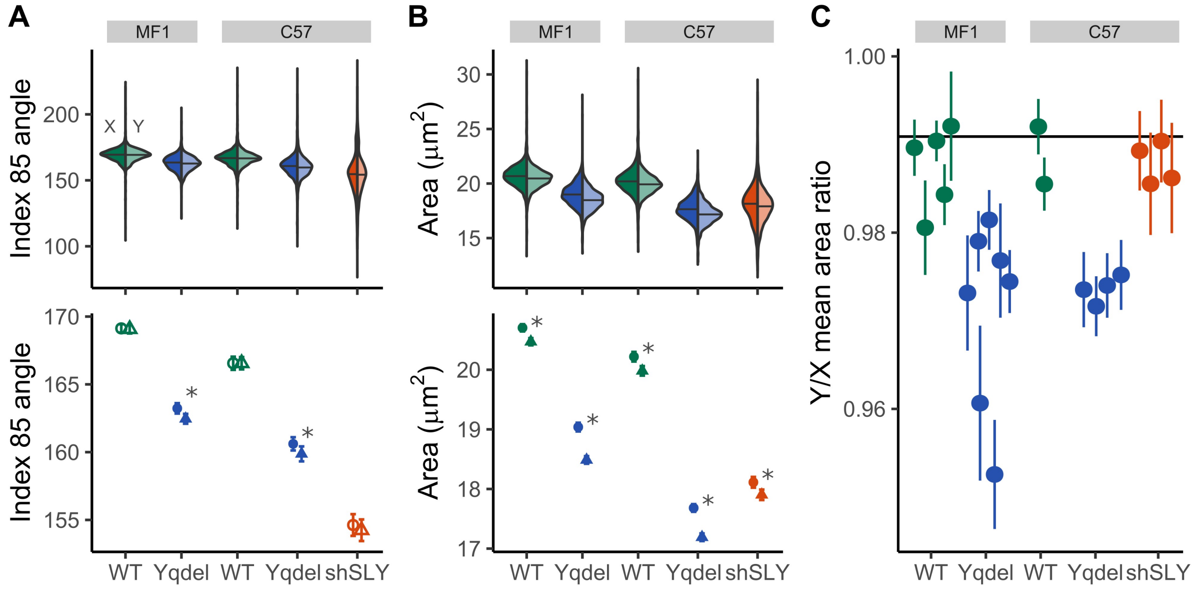

Figure S2. Detailed differences in sperm shape and size. Related to Figure 2. A) Angles at profile index 85 in each strain, comparing X- and Y-bearing sperm. The upper panel shows violin plots of the complete population data for each strain, while the lower panel shows the mean and standard error of the mean in order to highlight differences. Significant XY differences (Mann-Whitney test, $p < 0.05$) are marked '*'. Y-bearing sperm have a significantly more curved acrosome in Yqdel animals only. B) Comparison of nuclear areas, using the same format as (A); Y-bearing sperm are consistently smaller than X-bearing sperm in all genotypes including wild types. C) Comparison of the mean Y/X area ratio in each sample (see Table S1 for the details of sample numbers analysed per genotype), with standard error of the mean. The horizontal line indicates the mean wild type ratio. Wild type and shSLY samples have similar area ratios, while Yqdel samples show a further reduction in Y area.

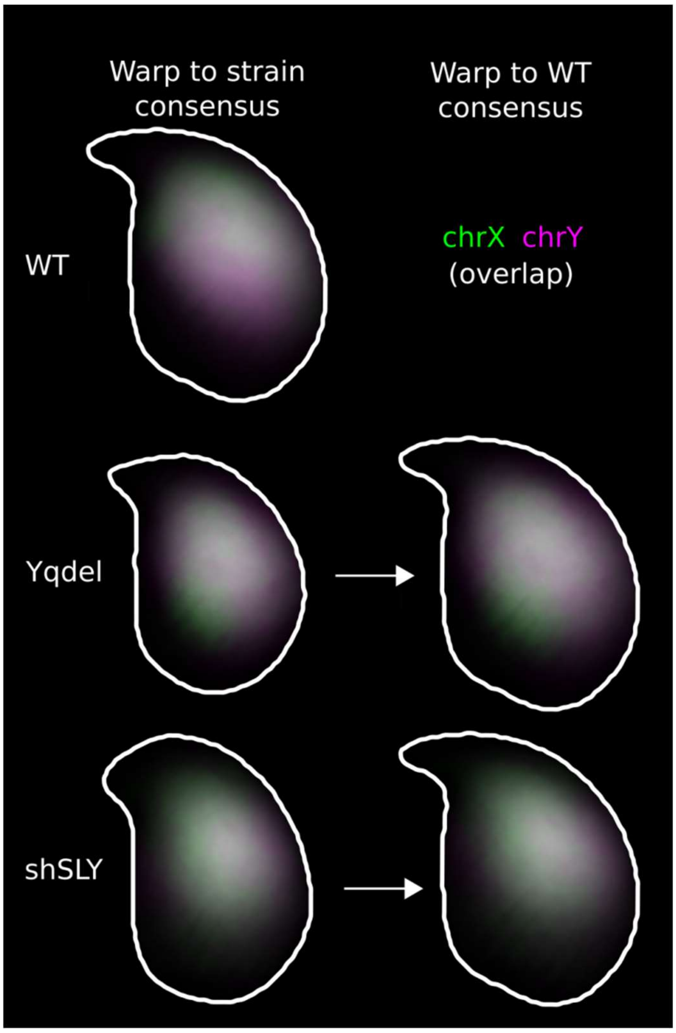

Figure S3. Comparison of chromosome positions in WT versus Yq-del sperm. Related to Figure 2. Chromosome territories for X and Y are sub-acrosomal in C57Bl6, and strongly overlap in all genotypes analysed (MS-SSIM* 0.86-0.94). Following warping to a WT template, the location of the X and Y chromosomes in sperm from Yqdel and shSLY males closely resembles their locations in wild type C57Bl6 sperm (MS-SSIM* 0.82-0.86). This indicates morphological changes are external to the nucleus, and do not involve other chromatin reorganisation.

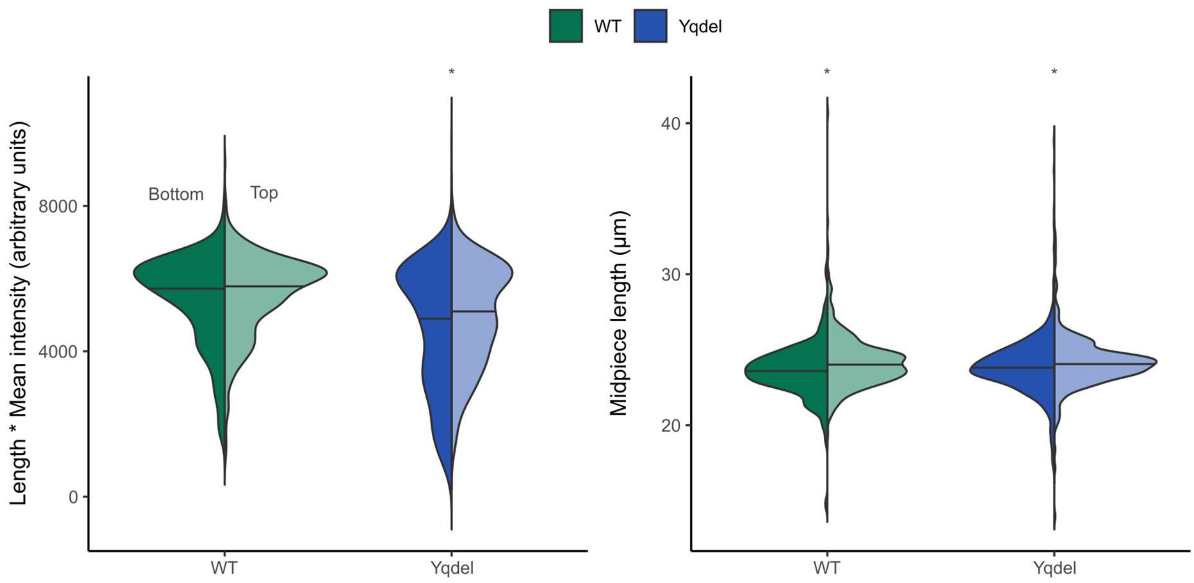

Figure S4. Poorer swimming sperm have lower mitochondrial activity. Related to Figure 4. Midpiece length multiplied by mean signal is a proxy for total mitochondrial activity. Horizontal lines show sample medians. A two-sample two-tailed Kolmogorov-Smirnov test detects no difference between top and bottom for multiplied values in wild type XY^{RIII} (p=0.73) and a significant difference in XY^{RIII}qdel (p=7.15E-05). Midpiece length is a proxy for mitochondrial count, and is slightly higher in top fractions in both XY^{RIII} (p=0.0004) and XY^{RIII}qdel (p=0.006).

Protocol	Paternal genotype	Number of males	Sperm status	Maternal details	Number of offspring	Percent female offspring	S.E.P.
Colony data (Cambs)	XYRIIIqdel	--	Pair or trio mating cages	Predominantly MF1	723	54.9%	1.85%
Colony data (Cambs)	XYRIII	--	Pair or trio mating cages	Predominantly MF1	922	47.2%	1.64%
Colony data (Kent)	XYRIIIqdel	--	Pair or trio mating cages	MF1	526	58.9%	2.15%
Colony data (Kent)	XYRIII	--	Pair or trio mating cages	MF1	534	45.7%	2.16%
Colony data (France)	XYRIIIqdel	--	Pair or trio mating cages	>80% C57Bl6/N	132	61.4%	4.24%
Colony data (France)	XYRIII	--	Pair or trio mating cages	>80% C57Bl6/N	151	51.7%	4.07%
Colony data (France)	shSLY	--	Pair or trio mating cages	>80% C57Bl6/N	16	87.5%	8.27%
IVF, GFP scoring of blastocysts	XGFPYRIIIqdel	6	cauda epididymal sperm retrieved for IVF	CBAB6 F1 oocytes (cumulus retained)	243	44.4%	3.19%
				CBAB6 F1 oocytes (cumulus removed)	203	44.8%	3.49%
				Total:	446	44.6%	2.35%
Superovulation, natural mating, 2-cell embryo recovery, GFP scoring of blastocysts	XGFPYRIIIqdel	9	fresh (3 day mating interval)	CBAB6 F1	173	55.5%	3.78%
			aged (14+ day mating interval)	CBAB6 F1	251	53.8%	3.15%

		4	aged (14+ day mating interval)	MF1	87	60.9%	5.23%
				Total:	511	55.6%	2.20%
Superovulation, natural mating, 2-cell embryo recovery, GFP scoring of blastocysts	XGFPYRIII	9	fresh (3 day mating interval)	CBAB6 F1	150	50.0%	4.08%
			aged (14+ day mating interval)	CBAB6 F1	104	47.1%	4.89%
		4	aged (14+ day mating interval)	MF1	73	38.4%	5.69%
				Total:	327	46.5%	2.76%
Natural mating (timed oestrus), GFP scoring of mid-gestation embryos	XGFPYRIIIqdel	7	~7 day mating interval	XYRIII daughters	143	58.0%	4.13%
			~7 day mating interval	XYRIIIqdel daughters	159	54.7%	3.95%
				Total:	302	56.3%	2.85%
Natural mating (timed oestrus), GFP scoring of mid-gestation embryos	XGFPYRIII	7	~7 day mating interval	XYRIII daughters	111	43.2%	4.70%
			~7 day mating interval	XYRIIIqdel daughters	143	48.3%	4.18%
				Total:	254	46.1%	3.13%

Table S1. Offspring sex ratios in animals in this study. Related to Figure 1. Offspring sex ratios shift in favour of females under natural mating in animals carrying the Y^{RIII}qdel chromosome compared to animals carrying the Y^{RIII} chromosome when using both fresh and aged sperm; this difference is abolished by IVF using embryos with or without cumulus cells (see also **Figure 1**). S.E.P, standard error of proportion.

		WT		Yqdel		shSLY	
**Background**	**Cluster**	**#X / #Y sperm**	**%X ± S.E.P.**	**#X / #Y sperm**	**%X ± S.E.P.**	**#X / #Y sperm**	**%X ± S.E.P.**
MF1	N1	995 / 946	51.3 ± 1.1%	68 / 47	59.1 ± 4.6%	-	-
	N2	1114 / 1022	52.2 ± 1.1%	135 / 113	54.4 ± 3.2%	-	-
	A1	35 / 24	59.3 ± 6.4%	1015 / 1053	49.1 ± 1.1%	-	-
	A2	53 / 49	52 ± 4.9%	420 / 568	42.5 ± 1.6%	-	-
	**Total**	**2197 / 2041**	**51.8 ± 0.8%**	**1638 / 1781**	**47.9 ± 0.9%**	-	-
C57Bl6	N	969 / 961	50.2 ± 1.1%	65 / 47	58 ± 4.7%	2 / 1	66.7 ± 27.2%
	A1	58 / 46	55.8 ± 4.9%	808 / 776	51 ± 1.3%	33 / 26	55.9 ± 6.5%
	A2	20 / 21	48.8 ± 7.8%	190 / 217	46.7 ± 2.5%	198 / 161	55.2 ± 2.6%
	A3	17 / 18	48.6 ± 8.4%	47 / 48	49.5 ± 5.1%	943 / 963	49.5 ± 1.1%
	B1	194 / 189	50.7 ± 2.6%	374 / 356	51.2 ± 1.9%	346 / 321	51.9 ± 1.9%
	B2	104 / 119	46.6 ± 3.3%	220 / 203	52 ± 2.4%	290 / 295	49.6 ± 2.1%
	S	30 / 30	50 ± 6.5%	155 / 122	56 ± 3%	669 / 690	49.2 ± 1.4%
	**Total**	**1392 / 1384**	**50.1 ± 0.1%**	**1859 / 1769**	**51.2 ± 0.1%**	**2481 / 2457**	**50.2 ± 0.1%**

Table S2. Number and proportion of X- and Y-bearing sperm in each morphological cluster. Related to Figure 3. On an MF1 background, more abnormal sperm are enriched for Y-bearing sperm (Fisher's exact test, p=0.002) in Yqdel samples, but not in WT samples (p=1). On the C57Bl6 background, a similar gradient is observed in Yqdel and shSLY samples, but is not statistically significant in these (p=0.63, 0.66), or in WT (p=1). S.E.P, standard error of proportion.

Type	Sample	Number of animals	Ages (weeks)	n cells	X	Y	Average X area	Average Y area	Y/X area ratio	Area difference	S.E.M.	Volume difference	S.E.M.
**MF1 WT**	WT P1	8	13 - 16	1219	634	585	20.78	20.56	99.0%	1.0%	0.3%	1.6%	0.5%
	WT P2	8	10 - 14	777	426	351	20.37	20.05	98.4%	1.6%	0.3%	2.3%	0.5%
	WT I1	1	10.9	1524	777	747	20.93	20.73	99.0%	1.0%	0.2%	1.4%	0.3%
	WT I2	1	29.7	305	150	155	20.76	20.59	99.2%	0.8%	0.6%	1.2%	0.9%
	WT I3	1	26.1	413	210	203	20.28	19.88	98.1%	1.9%	0.5%	2.9%	0.8%
	Average						*20.62*	*20.36*	*98.7%*	*1.3%*	*0.2%*	*1.9%*	*0.3%*
	**Aggregate**			4238	2197	2041	**20.70**	**20.47**	**98.9%**	**1.1%**	**0.2%**	**1.7%**	**0.2%**
**MF1 Yqdel**	Yqdel P1	5	11 - 14.5	904	386	518	18.50	18.02	97.4%	2.6%	0.4%	3.8%	0.5%
	Yqdel P2	6	9.3	308	119	189	18.93	18.03	95.3%	4.7%	0.6%	7.0%	0.9%
	Yqdel I1	1	11	860	440	420	19.76	19.35	97.9%	2.1%	0.3%	3.1%	0.5%
	Yqdel I2	1	11	262	136	126	18.79	18.35	97.7%	2.3%	0.6%	3.5%	1.0%
	Yqdel I3	1	11	574	279	295	18.93	18.58	98.1%	1.9%	0.3%	2.8%	0.5%
	Yqdel I4	1	31.3	177	91	86	19.31	18.55	96.1%	3.9%	0.9%	5.8%	1.3%
	Yqdel I5	1		187	94	93	18.44	17.94	97.3%	2.7%	0.7%	4.0%	1.0%

	Average						*18.95*	*18.40*	*97.1%*	*2.9%*	*0.4%*	*4.3%*	*0.5%*
	**Aggregate**			3419	1638	1781	**19.01**	**18.49**	**97.3%**	**2.7%**	**0.2%**	**4.1%**	**0.3%**
**C57Bl6 WT**	WT 1	1	24.6	1541	784	757	20.30	20.01	98.6%	1.4%	0.3%	2.2%	0.5%
	WT 2	1	24.6	1235	608	627	20.11	19.95	99.2%	0.8%	0.3%	1.2%	0.5%
	Average						*20.21*	*19.98*	*98.9%*	*1.1%*	*0.8%*	*1.7%*	*1.1%*
	**Aggregate**			2776	1392	1384	**20.22**	**19.98**	**98.8%**	**1.2%**	**0.2%**	**1.8%**	**0.3%**
**C57Bl6 Yqdel**	Yqdel 1	1	14.6	1102	566	536	17.94	17.47	97.4%	2.6%	0.4%	3.9%	0.5%
	Yqdel 2	1	14.6	1101	594	507	17.87	17.36	97.2%	2.8%	0.3%	4.2%	0.5%
	Yqdel 3	1	11.4	690	344	346	17.60	17.14	97.4%	2.6%	0.4%	3.9%	0.6%
	Yqdel 4	1	11.4	735	355	380	17.04	16.62	97.5%	2.5%	0.4%	3.7%	0.6%
	Average						*17.61*	*17.15*	*97.4%*	*2.6%*	*0.1%*	*3.9%*	*0.1%*
	**Aggregate**			3628	1859	1769	**17.68**	**17.19**	**97.2%**	**2.8%**	**0.2%**	**4.1%**	**0.3%**
**C57Bl6 shSLY**	shSLY 1	1	14.9	1149	587	562	17.89	17.65	98.6%	1.4%	0.6%	2.1%	0.9%
	shSLY 2	1	24.6	1261	653	608	17.84	17.65	98.9%	1.1%	0.5%	1.6%	0.7%
	shSLY 3	1	24.6	1368	666	702	17.99	17.81	99.0%	1.0%	0.5%	1.4%	0.7%

	shSLY 4	1	24.6	1160	575	585	18.80	18.53	98.6%	1.4%	0.6%	2.2%	0.9%
	Average						*18.13*	*17.91*	*98.8%*	*1.2%*	*0.1%*	*1.8%*	*0.2%*
	**Aggregate**			4938	2481	2457	**18.11**	**17.90**	**98.8%**	**1.2%**	**0.3%**	**1.7%**	**0.4%**

Table S3. Details of the individual samples analysed, and the measured size differences in X- and Y- bearing sperm. Related to Figure 2 and Figure 3. Details of the individual samples analysed, and the measured size differences in X- and Y- bearing sperm. S.E.M, standard error of the mean. The volume difference was calculated from area, assuming an equivalent reduction in sperm thickness.

	Estimate	Std. Error	z value	Pr(>|z|)
(Intercept)	0.015374	0.018551	0.829	0.407
Segment	-0.010992	0.004764	-2.307	0.021
Strain alone	0.038406	0.026236	1.464	0.143
Live/dead state	-0.142011	0.024665	-5.758	8.53E-09
Segment:Strain	-0.001866	0.006737	-0.277	0.782
Segment:State	0.032999	0.006333	5.211	1.88E-07
Strain:State	0.231879	0.034883	6.647	2.98E-11
Segment:Strain:State	-0.049502	0.008956	-5.527	3.25E-08

Table S4. Summary of the beta regression on swim-up data, showing the significance of each parameter and their interactions. Related to Figure 4. Summary of the beta regression on swim-up data, showing the significance of each parameter and their interactions. The pseudo R^2 was 0.87.
